# Leptin alters energy intake and fat mass but not energy expenditure in lean subjects

**DOI:** 10.1038/s41467-020-18885-9

**Published:** 2020-10-13

**Authors:** Pavlina Chrysafi, Nikolaos Perakakis, Olivia M. Farr, Konstantinos Stefanakis, Natia Peradze, Aleix Sala-Vila, Christos S. Mantzoros

**Affiliations:** 1 Department of Medicine, Beth Israel Deaconess Medical Center, Harvard Medical School, Boston, MA 02215 USA; 2 Barcelonaβeta Brain Research Center, Pasqual Maragall Foundation, Barcelona, Spain; 3 Institut Hospital del Mar d’Investigacions Mèdiques (IMIM), Barcelona, Spain

**Keywords:** Metabolism, Endocrine system and metabolic diseases

## Abstract

Based on studies in mice, leptin was expected to decrease body weight in obese individuals. However, the majority of the obese are hyperleptinemic and do not respond to leptin treatment, suggesting the presence of leptin tolerance and questioning the role of leptin as regulator of energy balance in humans. We thus performed detailed novel measurements and analyses of samples and data from our clinical trials biobank to investigate leptin effects on mechanisms of weight regulation in lean normo- and mildly hypo-leptinemic individuals without genetic disorders. We demonstrate that short-term leptin administration alters food intake during refeeding after fasting, whereas long-term leptin treatment reduces fat mass and body weight, and transiently alters circulating free fatty acids in lean mildly hypoleptinemic individuals. Leptin levels before treatment initiation and leptin dose do not predict the observed weight loss in lean individuals suggesting a saturable effect of leptin. In contrast to data from animal studies, leptin treatment does not affect energy expenditure, lipid utilization, SNS activity, heart rate, blood pressure or lean body mass.

## Abstract (web summary)

Leptin treatment is effective to reduce body weight in animal models, but patients with obesity and associated hyperleptinemia do not respond well to leptin therapy. Here the authors report a retrospective analysis of four clinical trials in normo- and mildly hypoleptinemic individuals and show that leptin therapy alters food intake in the short term and reduces weight and fat mass in the long term without effects on energy expenditure.

## Introduction

Leptin, the prototypical adipokine, circulates at levels proportional to fat mass^1,2^ and responds to acute changes in energy intake^3–5^.

On the basis of experiments in ob/ob and lean mice, leptin was thought to effectively cause weight loss by regulating appetite, energy expenditure, sympathetic nervous system (SNS) activity, lipolysis, and lipid–carbohydrate utilization^6–8^. In contrast, leptin administration has been far less effective in animal models of obesity with leptin excess^9^. In humans, with the exception of severe leptin deficiencies due to leptin mutations (congenital leptin deficiency, CLD) or lipodystrophies (generalized (GL) or partial lipodystrophies (PL))^10–13^, the majority of studies in overweight or obese populations with hyperleptinemia showed minimal if any effects of leptin treatment on weight or body composition^14–22^. Recent research efforts focus therefore on the identification of threshold leptin concentrations in the blood, below which a treatment with leptin may be effective by leading to significant weight loss among people with obesity^23^. Additionally, due to the poor efficacy of leptin treatments on weight regulation in obesity, it has been questioned whether leptin does act to reduce body weight in humans, emphasizing the need to investigate the physiology of leptin and its effects on metabolic outcomes in lean individuals who may be more likely to respond to leptin administration^24,25^.

**Fig. 1:**
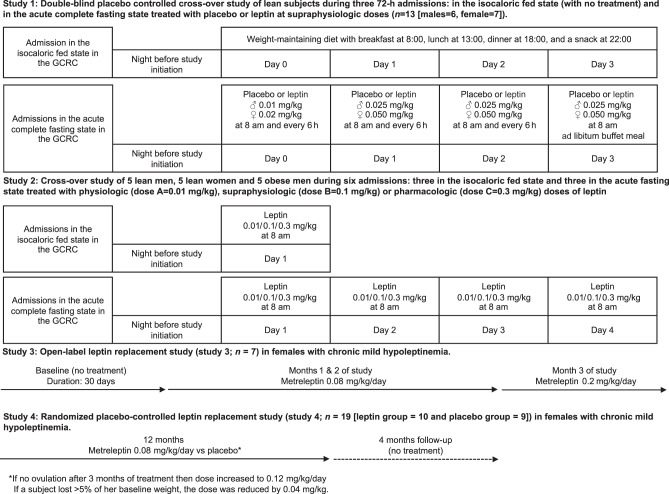
Schematic representation of the study designs of the four clinical studies. Study 1: Eight healthy lean men and seven healthy lean women were studied under three separate Clinical Research Center (CRC)-based conditions for 72 h: one under isocaloric fed state conditions (normoleptinemia) and two during complete fasting state conditions (induced hypoleptinemia) scheduled in a random order and in a double-blind fashion with administration of physiologic replacement leptin doses (fasting + leptin) or placebo (fasting + placebo). Study 2: Five lean men, five men with obesity, and five lean women participated in three fed-normoleptinemic and three fasting-induced hypoleptinemic studies, which were conducted in the CRC, with leptin administration at three different doses (Dose A = 0.01 mg/kg, Dose B = 0.1 mg/kg, Dose C = 0.3 mg/kg). Study 3: Open-label long-term leptin treatment in mildly hypoleptinemic women. Study 4: Placebo-controlled long-term leptin treatment in mildly hypoleptinemic women.

We have previously reported no effect of short-term leptin treatment on weight loss during acute 72-h fasting (studies 1 and 2) (Fig. 1 for study design)^26,27^. In contrast, we observed a significant reduction of weight in lean chronic and partial hypoleptinemic women due to strenuous exercise (studies 3 and 4) treated with leptin from 8 and up to 36 weeks (Fig. 1 for study designs)^28,29^. Here, we perform new measurements and analyze data from our previous studies^26–30^ to (1) assess whether circulating concentrations of leptin before treatment initiation are associated with the weight loss observed in our study subjects, (2) investigate whether any effects of leptin either in the short term during fasting and/or in the fed state are dose-dependent, by employing physiological vs supraphysiological vs pharmacological doses of leptin, and explore whether such effects may differ between lean men, lean women, and obese men, (3) investigate the trajectories of weight and fat mass changes in relation to leptin levels during long-term leptin treatment and after its termination, and (4) determine the potential underlying mechanistic pathways through which leptin affects the physiology of energy homeostasis in lean subjects by testing energy intake (measured caloric intake using ad libitum feeding), energy expenditure (physical activity and resting metabolic rate (RMR) measurements), SNS outputs (heart rate (HR), blood pressure (BP), catecholamine levels, and the renin–angiotensin–aldosterone system (RAAS)), fuel utilization, and metabolite–lipid–lipoprotein profile.

## Results

### Baseline leptin levels do not predict weight-loss magnitude

**Fig. 2:**
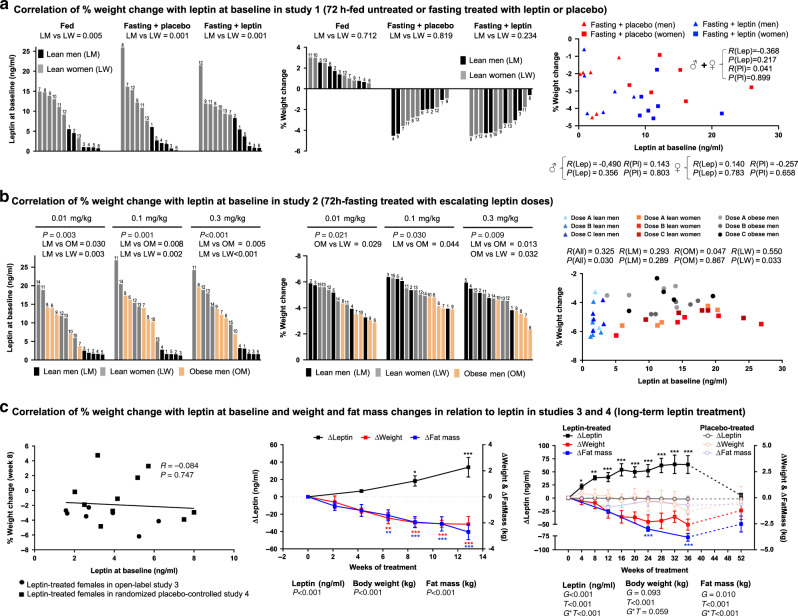
Leptin effects on weight and fat mass. **a** Cross-over study of lean subjects during 72-h fed state, fasting+placebo and fasting+leptin (study 1; *n* = 13). Left: baseline leptin levels in each admission. Center: % weight change at the end of each admission. Right: correlation of baseline leptin with % weight change at the end of each admission. Numbers above bars correspond to subject ID. *P* values of unpaired *t* test between lean men (LM) vs lean women (LW) and of correlations are reported; *R*, correlation coefficient. **b** Cross-over study of LM, LW, and obese men (OM) in 72-h fasting treated with escalating leptin doses (study 2; *n* = 15). Left: baseline leptin levels in each admission, Center: % weight change at the end of each admission. Right: correlation of baseline leptin with % weight change at the end of each admission. Numbers above bars correspond to subject ID. P values from one-way ANOVA, from post hoc Bonferroni test between LM vs LW vs OW and from correlations are reported. **c** Open-label (study 3; *n* = 7) and placebo-controlled long-term leptin treatment study (study 4; *n* = 19 (leptin = 10; placebo = 9)) in women with mild hypoleptinemia. Left: correlation of baseline leptin with % weight change after 8 weeks of leptin treatment. Subjects of study 3 were combined with leptin-treated subjects of study 4 in one analysis. Center and right: changes of leptin, body weight, and fat mass from baseline (Δ = change from baseline at each timepoint). In study 4, dashed lines correspond to the washout period after 36 weeks of study. In study 3, *P* values (*P*) for time effect (i.e., days of treatment) and in study 4, *P* values of *G* (group: leptin or placebo), *T* (time: weeks of treatment), and G*T interaction of mixed models adjusted for baseline are reported. By *P* < 0.05 (study 3) and G*T < 0.05 (study 4), post hoc Bonferroni test was performed (only significant results are reported). One, two, or three asterisks indicate *P* < 0.05, <0.01, or <0.001 for the specific timepoint vs baseline in study 3 and for leptin vs placebo in the specific timepoint in study 4. Correlations were performed with Pearson’s or Spearman’s correlation test. Data are presented as means ± SEMs. Exact *P* values**: b**, left: leptin at baseline dose 0.3 mg/kg = 0.003 × 10^−1^; leptin at baseline dose 0.3 mg/kg LM vs LW = 0.003 × 10^−1^. **c** Center**:** leptin = 0.003 × 10^−1^; weight = 0.001 × 10^−3^; fat mass = 0.002 × 10^−5^; leptin post hoc test at 8_1/2_ weeks = 0.031 and at 13 weeks = 0.003 × 10^−1^. Weight post hoc test at 6_1/2_ weeks = 0.002, at 8_1/2_ weeks = 0.001 × 10^−1^, at 11 weeks = 0.001 × 10^−1^, and at 13 weeks = 0.001 × 10^−1^. Fat mass post hoc test at 6_1/2_ weeks = 0.002, at 8_1/2_ weeks = 0.002 × 10^−2^, at 11 weeks = 0.005 × 10^−3^, and at 13 weeks = 0.004 × 10^−4^. **c**, Right**:** leptin *G* = 0.005 × 10^−2^; *T* = 0.007 × 10^−5^; G*T = 0.002 × 10^−5^; body weight *T* = 0.007 × 10^−5^; fat mass *T* = 0.009 × 10^−5^; G*T = 0.001 × 10^−2^; leptin post hoc test at 4 weeks = 0.039; at 8 weeks = 0.001; at 12 weeks = 0.004 × 10^−1^; at 16 weeks = 0.005 × 10^−3^; at 20 weeks = 0.003 × 10^−2^; at 24 weeks = 0.001 × 10^−2^; at 28 weeks = 0.002 × 10^−3^; at 32 weeks = 0.004 × 10^−3^; at 36 weeks = 0.002 × 10^−3^. Fat mass post hoc test at 24 weeks = 0.002 × 10^−1^; at 36 weeks = 0.001 × 10^−1^.

In study 1 (three interventions: fed—untreated, fasting—treated with leptin, and fasting—treated with placebo) and study 2 (six interventions: fed and fasting state treated with physiological, supraphysiological, or pharmacological doses of leptin) (see Fig. 1 for study designs), lean men had consistently lower levels of leptin before each intervention compared to lean women, whereas obese men had similar leptin levels to lean women (study 2) (Fig. 2a, b, left and Supplementary Figs. 2 and 3). In both studies and across all interventions, the % body weight change was similar between lean men and women (Fig. 2a, b, middle) and lower in obese men (Fig. 2b, middle). In study 1, when men and women were investigated both together and separately, there was no association or trend (all *P* values > 0.2) between leptin levels in the blood at baseline (before treatment initiation) (Fig. 2a, right) and % weight loss after 72-h fasting treated with placebo or leptin. Similarly, in study 2, although the sample size per group was small (*n* = 5), no strong association of leptin levels at baseline with % weight loss was observed for obese and lean men, when values from all doses were combined, both before (Fig. 2b, right) and after adjusting for intraindividual variability (i.e., for contribution of three values in the correlation by each subject) (Supplementary Fig. 1a, left and middle). For lean women, there was an association of lower % weight loss with higher baseline leptin values (with no clear cut-off levels) before (Fig. 2b, right) but not after adjusting for intraindividual variability (Supplementary Fig. 1a, right). Finally, the % weight loss was not affected by leptin dose in any of the three groups (lean men, lean women, and obese men) (Supplementary Fig. 1b).

In studies 3 and 4 (long-term leptin replacement in mildly hypoleptinemic women, see Fig. 1 for study design), women had generally low body fat % (mean with min, max % for study 3 = 22.7 (17.7, 28), study 4 = 22.4 (14.3, 29.9)) but they all had stable weight in the last 6 months prior to study participation. Additionally, none of the women in study 3 and only three women all from the placebo group in study 4 were underweight (BMIs 17.6, 18.1, and 18.4 kg/m^2^). Leptin levels of the women in studies 3 and 4 were low (mean ± SE, study 3 = 3.4 ± 0.7, study 4 = 4.3 ± 0.4 ng/ml), but generally higher than the leptin levels of patients with generalized lipodystrophy (1.3 ± 0.3 ng/ml)^31^, thus representing a model of milder, acquired partial leptin deficiency.

Baseline leptin levels before treatment initiation spanned in both studies between 1.5 and 8 ng/ml. In agreement with our observations in the short-term fasting studies, baseline leptin levels were not associated with % weight loss during the first 8 weeks of leptin treatment (Fig. 2c, left), during which women both in studies 3 and 4 were treated with the same dose of leptin (0.08 mg/kg/day). In contrast, weight and fat mass decreased in parallel in response to the increasing levels of leptin in both studies (Fig. 2c, middle and right). In study 4, the weight changes were stabilized on week 12, when leptin dose adjustments per study protocol occurred in order to prevent further weight loss, whereas interestingly fat mass loss continued up to the 36th week (Fig. 2c, right). Of note, both in studies 3 and 4, no lean mass loss was observed (Supplementary Fig. 1c, d). Weight and fat mass partially reverted toward baseline after discontinuation of leptin treatment and return of leptin to pretreatment levels (Fig. 2c, right).

Altogether, across multiple studies, baseline leptin did not predict the % weight loss observed after leptin administration in lean populations. Lean men and women demonstrate similar % weight loss during acute fasting, which is independent from leptin dose. Obese men show less % weight loss compared to lean individuals during acute fasting, which is, similar to lean population, independent from baseline leptin levels and leptin dose. During long-term leptin treatment, the increasing leptin levels in women with partial acquired hypoleptinemia are associated with a parallel reduction of fat mass and consequently weight, which are both reversible after treatment termination. Next, we aimed to investigate how leptin regulates body weight in lean individuals by investigating the effects of leptin on parameters related to energy intake, energy expenditure, lipolysis, and lipid utilization.

### Leptin affects energy intake but not energy expenditure

**Fig. 3:**
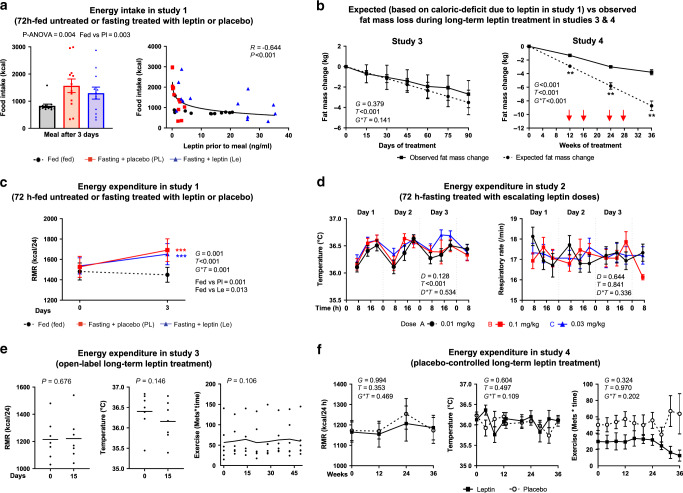
Short- and long-term leptin effects on energy intake and expenditure. **a** Energy intake after 72-h in fed state, fasting+placebo, or fasting + leptin (study 1, *n* = 13). *P* values from repeated measure ANOVA and post hoc Bonferroni test are reported. R, correlation coefficient. **b** Expected (based on leptin-induced caloric deficit in study 1) vs observed fat mass loss during long-term leptin treatment in studies 3 and 4. As per the protocol, in study 4, if a subject lost >5% of baseline weight, the dose was reduced by 0.04 mg/kg (red arrows). *P* values of *G* (group: observed or expected fat mass change), *T* (days/weeks of treatment), and G*T interaction of mixed models are reported. By G*T < 0.05, post hoc Bonferroni test was performed: two asterisks indicate *P* < 0.01 for observed vs expected fat mass change at the specific timepoint. **c** Energy expenditure during 72-h fed state, fasting+ placebo, and fasting + leptin (study 1, *n* = 13). *P* values of *G* (group: fed, fasting + placebo, fasting + leptin), *T* (days of treatment), and G*T interaction of mixed models adjusted for baseline are reported. Post hoc Bonferroni test was performed between the estimated means of the three groups and between the three groups at each timepoint. Three asterisks indicate *P* < 0.001 for fed vs fasting + placebo (red) and fed vs fasting + leptin (blue). **d** Energy expenditure during 72-h fasting treated with escalating leptin doses (study 2, *n* = 15). *P* values of *D* (dose: 0.01, 0.1, and 0.3 mg/kg/d), *T* (hours of fasting), and D*T interaction of mixed models adjusted for baseline are reported. **e** Energy expenditure during open-label long-term leptin treatment in mildly hypoleptinemic women (study 3, *n* = 7). *P* values of paired *t* test (RMR and body temperature) and of time effect of mixed models adjusted for baseline (exercise score) are reported. No post hoc test was performed since *P* > 0.05. **f** Energy expenditure during placebo-controlled long-term leptin treatment in mildly hypoleptinemic women (study 4, *n* = 19 (leptin = 10; placebo = 9)). *P* values of *G* (group: placebo or leptin), *T* (weeks of study), and G*T interaction of mixed models adjusted for baseline are reported. No post hoc test was performed since G*T > 0.05. All *P* values are two-sided. For post hoc Bonferroni test, only significant results are reported. Data are demonstrated as means ± SEMs. Exact *P* values: **a** Correlation of food intake with leptin prior to meal = 0.002 × 10^−2^. **b** Study 4**:** post hoc test at 12 weeks = 0.002; at 24 weeks = 0.001; at 36 weeks = 0.003. **c** Day 3 fed vs Pl = 0.004 × 10^−3^; fed vs Le = 0.003 × 10^−1^. **d** Temperature *T* = 0.002 × 10^−12^.

The lack of a weight-regulatory effect by leptin treatment during short-term fasting in lean individuals indicates no impact of leptin on energy balance in the short term, and when any potential effects of leptin on energy intake are experimentally controlled with the imposed fasting. To assess now the effects of leptin on energy intake in this experimental setting, an ad libitum meal was offered at the end of the 72-h fasting treated with placebo or leptin, as well as at the end of a 72-h isocaloric fed state. In this meal, higher caloric intake was observed after fasting treated with placebo compared to fed state, but this was partially reduced (−17.3%) and was closer to fed-state levels after fasting treated with leptin (Fig. 3a, left). Furthermore, leptin levels directly before meal intake correlated negatively, curvilinearly, and strongly with calorie intake (*r* = −0.644, *P* < 0.001, Fig. 3a right), with an inflection point approximately at 10 ng/ml, when all values from the three interventions were combined (unadjusted), as well as when we have adjusted for treatment with placebo or leptin during fasting (after adjustment for intra-subject variability, *r* = −0.569, *P* = 0.042, Supplementary Fig. 1e).

In the long-term leptin studies, we have not performed an ad libitum meal to assess food intake. Nevertheless, presuming that the ~18% caloric deficit during a test meal that we detected with leptin treatment compared to placebo during short-term fasting in women of study 1 persists with long-term leptin administration in women of studies 3 and 4, we have projected the expected fat mass loss due to reduced energy intake and compared it with the observed (true) fat mass loss during treatment. For these projections, we have used information about the average food intake of the subjects in studies 3 and 4 that were collected by self-report questionnaires at screening. In study 3, where leptin dose was increased in women with no ovulation after 2 months of treatment from 0.08 mg/kg/day to 0.2 mg/kg/day and was not adjusted according to weight changes, the expected fat mass loss due to reduced energy intake is almost identical to the observed one (Fig. 3b, left). In study 4, total fat mass was assessed for the first time after 3 months of leptin treatment in a stable dose, which was much lower in the third month compared to the dose of study 3. Additionally, in study 4, the increase of leptin dose after the third month of treatment was much smaller compared to study 3, whereas in many participants, a dose reduction was necessary in order to prevent further weight loss per study protocol. Given the expected plateauing of leptin effects with time, as with most weight loss treatments, a smaller fat mass loss is observed than the one expected based on the ~18% daily caloric deficit created by leptin, but subjects in the leptin treatment group still lost 1.3 kg of fat at 12 weeks, 3.0 kg at 24 weeks, and 3.8 kg of fat up to 36 weeks of leptin treatment (Fig. 3b, right).

Next, we assessed whether markers of energy expenditure are affected by leptin treatment. RMR and/or body temperature did not change: (a) by short-term leptin treatment compared to placebo in lean people during acute fasting (Fig. 3c, study 1), (b) by escalating doses of leptin during acute fasting or in the fed state in lean men, lean women, or obese men (Fig. 3d and Supplementary Figs. 2, 3, study 2)^32^, and (c) by long-term leptin treatment in lean mildly hypoleptinemic women (Fig. 3e, f, studies 3 and 4). Additionally, respiratory rate, which is a parameter that is included in most models of estimation of total energy expenditure based on respiratory function^33^, was not affected by escalating doses of leptin during acute fasting or in fed state (Fig. 3d and Supplementary Figs. 2, 3, study 2). Finally, physical activity in lean mildly hypoleptinemic women as assessed with metabolic equivalents*time was also not altered with leptin administration (Fig. 3e, f, studies 3 and 4).

### Leptin has no effect on markers of SNS activity

**Fig. 4:**
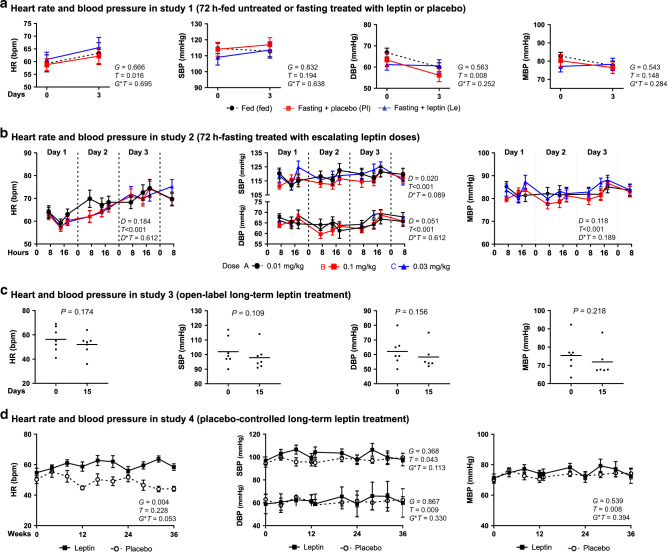
Leptin effects on heart rate and blood pressure. **a** Seventy-two hours fed state or fasting + leptin or fasting + placebo (study 1, *n* = 13). *P* values of *G* (group: fed or fasting + placebo or fasting + leptin), *T* (time: days of study), and G*T interaction of mixed models adjusted for baseline are reported. **b** Seventy-two hours fasting treated with escalating leptin doses (study 2, *n* = 15). *P* values of *D* (dose: 0.01 or 0.1 or 0.3 mg/kg/d), *T* (time: hours of fasting), and D*T interaction of mixed models adjusted for baseline are reported. **c** Open-label long-term leptin treatment in mildly hypoleptinemic women (study 3, *n* = 7). *P* value (*P*) of paired *t* test is reported. **d** Placebo-controlled long-term leptin treatment in mildly hypoleptinemic women (study 4, *n* = 19 (leptin = 10; placebo = 9)). *P* values of *G* (group: placebo or leptin), *T* (time: weeks of study), and G*T interaction of mixed models adjusted for baseline are reported. No post hoc Bonnferroni test was performed since G*T > 0.05 (studies 1 and 4) and D*T > 0.05 (study 2). All *P* values are two-sided. Data are demonstrated as means ± SEMs. Exact *P* values: **b** HR *T* = 0.001 × 10^−17^; SBP *T* = 0.003 × 10^−1^; DBP *T* = 0.002 × 10^−2^; MBP *T* = 0.002 × 10^−2^.

Several animal studies have demonstrated regulatory effects of leptin on SNS activity, which may also lead to changes in energy expenditure. We have thus analyzed data from several markers of SNS activity in our studies. During short-term fasting, leptin replacement did not change HR, systolic BP (SBP), and diastolic BP (DBP) (Fig. 4a). Similarly, no differences in HR, SBP, and DBP were observed during fasting or fed state between the different doses of leptin, when lean men, lean women, and obese men were investigated together (Fig. 4b) or when we have compared the values between them (Supplementary Figs. 2 and 3). Similar to the results in the short-term fasting studies, long-term leptin treatment in hypoleptinemic women did not affect HR, SBP, or DBP (Fig. 4c, d).

**Fig. 5:**
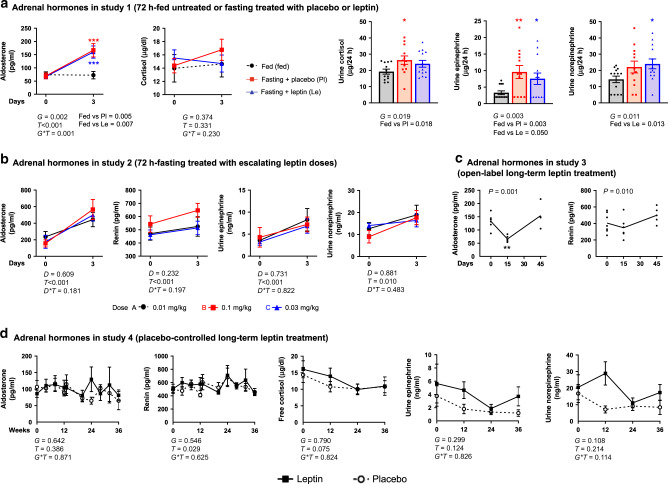
Leptin effects on adrenal hormones. **a** Seventy-two hours fed state or fasting+leptin or fasting + placebo (study 1, *n* = 13). Left: blood aldosterone and cortisol at the start and completion of the study. Right: 24-h urine cortisol, epinephrine, and norepinephrine collected at the last day of the study. *P* values of *G* (group: fed or fasting + placebo or fasting + leptin), *T* (time: days of study), and G*T interaction of mixed models, adjusted for baseline are reported. For urine catecholamines, *P* values were calculated with repeated measure ANOVA, since only the group factor existed. By G*T < 0.05 (blood aldosterone and cortisol) and by *G* < 0.05 (urine catecholamines), post hoc Bonferroni test was performed between the estimated means of the three groups and between the three groups at each timepoint. Three asterisks indicate *P* < 0.001 for fed vs fasting + placebo (red) and for fed vs fasting + leptin (blue) at the particular timepoint. **b** Seventy-two hours of fasting treated with escalating leptin doses (study 2, *n* = 15). *P* values of *D* (dose: 0.01 or 0.1 or 0.3 mg/kg/d), *T* (time: days of fasting), and D*T interaction of mixed models adjusted for baseline are reported. No post hoc Bonnferroni test was performed since D*T > 0.05. **c** Open-label long-term leptin treatment in mildly hypoleptinemic women (study 3, *n* = 7). *P* value (*P*) of time effect (i.e., days of study) of mixed models adjusted for baseline is reported. By *P* < 0.05 post hoc Bonferroni’s test for each timepoint compared to baseline was additionally performed and two asterisks indicate *P* < 0.01 for the specific timepoint vs 0 (baseline). **d** Placebo-controlled long-term leptin treatment in mildly hypoleptinemic women (study 4, *n* = 19 (leptin = 10; placebo = 9)). *P* values of *G* (group: placebo or leptin), *T* (time: weeks of study), and G*T interaction of mixed models adjusted for baseline are reported. No post hoc Bonnferroni test was performed since G*T > 0.05. For Bonferroni post hoc tests, only significant results are reported. All *P* values are two-sided. Data are demonstrated as means ± SEMs. Exact *P* values**: a** aldosterone *T* = 0.001 × 10^−3^; day 3 post hoc test for fed vs Pl = 0.004 × 10^−2^; fed vs Le = 0.006 × 10^−2^. **b** Aldosterone *T* = 0.001 × 10^−15^; renin *T* = 0.005 × 10^−17^; urine epinephrine *T* = 0.004 × 10^−1^. **c** Aldosterone post hoc test at 15 days = 0.005.

Higher SNS activity may also be resulting by increased adrenal function. During short-term fasting, aldosterone levels increased compared to fed state but independently from treatment (leptin or placebo) (Fig. 5a, left). Similarly, 24-h urine cortisol and catecholamines collected at the second day of the study were generally higher after fasting compared to fed state (Fig. 5a, right) but nonsignificantly different between leptin and placebo group (−8.5% for cortisol and +8.5% for norepinephrine in leptin treatment compared to placebo). In study 2, plasma aldosterone, renin, urine epinephrine, and norepinephrine increased during fasting (compared to baseline levels) at the same magnitude in all leptin doses (physiological, supraphysiological, or pharmacological) (Fig. 5b and Supplementary Fig. 2). Long-term leptin treatment in mildly hypoleptinemic women in study 3 resulted in a small, early coordinated decrease in aldosterone and renin during the first 15 days of treatment with return to baseline for both hormones at day 45 of treatment (Fig. 5c). In contrast to study 3, no decrease in aldosterone or renin was observed in study 4, and this was extended to a lack of changes in urine catecholamines and blood cortisol (Fig. 5d). Altogether, no robust evidence of significant changes on markers of SNS activity with leptin treatment was observed in our study populations.

### Long-term leptin transiently increases free fatty acids

Studies have suggested a differential role for leptin on lipid metabolism depending on energy status, with low leptin levels signaling the shift from carbohydrate to increased lipolysis and lipid utilization during starvation and with leptin treatment inducing lipolysis by stimulating SNS activity in nonfasting conditions^8,34,35^.

**Fig. 6:**
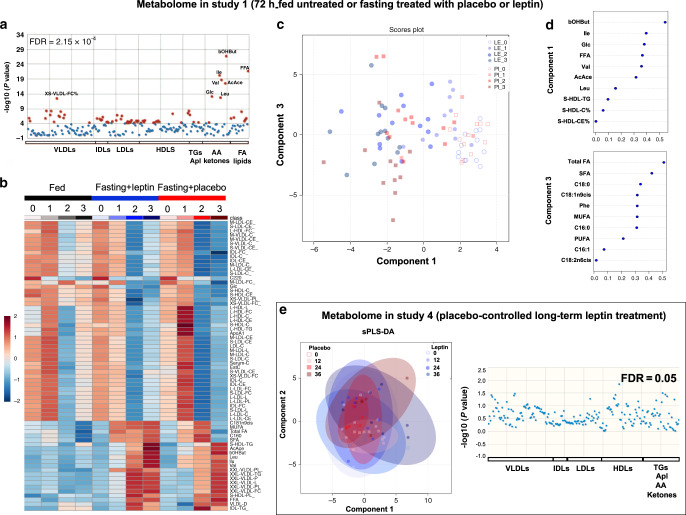
Metabolome changes with short- and long-term leptin treatment. **a**–**d** Effects on metabolite and lipid metabolism of 72-h fed state or fasting treated with leptin or placebo (study 1, *n* = 13). **a** Evaluation of metabolites, lipids, and lipoproteins with one-way ANOVA between the three admissions. Red dots indicate parameters significantly different and blue dots parameters not significantly different between groups (fed vs fasting + placebo vs fasting + leptin) with a preset false discovery rate of *P* < 2.15 × 10^−4^ (total 68 parameters significant). **b** Heatmap of the 68 significant parameters according to one-way ANOVA for the three admissions. **c** sPLS-DA analysis of fasting+leptin vs fasting + placebo: symbols indicate the measurement of component 1 in relation to measurement of component 3 for one subject/on one treatment/on one day of fasting: Blue circles correspond to leptin and red squares to placebo. Increasing color intensity indicates more time (days) of fasting. **d** The ten parameters that compose components 1 and 3 and their level of contribution (loading) to the component. **e** Effects on metabolite and lipid metabolism of long-term leptin treatment in mildly hypoleptinemic women (placebo-controlled study 4, *n* = 19 (leptin = 10; placebo = 9)). Left: sPLS-DA analysis of metabolites and lipoproteins in placebo vs leptin. Symbols indicate the measurement of component 1 in relation to component 2 for one subject/on one treatment/on one day of study: blue circles correspond to leptin and red squares to placebo. Increasing color intensity of symbol indicates more time (weeks) of study. Large oval-colored shapes indicate 95% confidence interval for each group. The observed major overlap between groups suggests no significant differences between placebo and leptin. Right: Evaluation of metabolites and lipoproteins with one-way ANOVA in placebo and leptin-treated subjects for up to 36 weeks. Each dot represents a parameter (blue dot = nonsignificant parameter, red = preset color for significant parameters but no such parameter was detected). NMR-based metabolomics were used to quantify amino acids, metabolites, and lipids bound to lipoproteins. GC/MS-EI was used to quantify fatty acid methyl esters. Le_0, Le_1 etc. indicate day 0 (baseline), 1 etc. of fasting + leptin. Pl_0, Pl_1 etc. indicate day 0 (baseline), 1 etc. of fasting + placebo. For metabolite nomenclature, see Supplementary Data 2.

In our lean population (study 1), respiratory quotient indicated a reduction in the utilization of carbohydrates and an increase in the utilization of lipids during short-term fasting, which was not affected by leptin treatment (Supplementary Fig. 4a). Similarly, in studies 3 and 4, no robust changes in macronutrient utilization during long-term leptin treatment were observed (Supplementary Fig. 4b, c). To further investigate the above finding, we have performed a metabolite–lipid–lipoprotein analysis. In study 1, 68 lipoproteins, lipids, and metabolites were significantly different between the three admissions (Fig. 6a, b). In a sparse partial least-squares discriminant analysis (sPLS-DA) between the two fasting conditions (leptin or placebo-treated), component 1 consisting of ten parameters discriminated progressively between the different days of fasting but not between placebo or leptin (in Fig. 6c, faint colors (days 0–1 of fasting) are gathered at the right (area of positive values for component 1) and bright colors (days 2–3 of fasting) at the left (area of negative values for component 1), whereas the circles (leptin) and the squares (placebo) are equally distributed from right to left). Component 1 included classic milestones of metabolic adaptation during starvation, such as amino acids and ketone bodies that their concentrations change with fasting but independent from treatment (placebo or leptin) (Supplementary Fig. 5a, b). This shows that blocking hypoleptinemia does not prevent the shift from carbohydrates to lipid utilization and ketone formation during starvation in lean humans. Similarly, in study 2, sPLS-DA demonstrated changes with time during fasting (component 2 consisting of ketone bodies, amino acids, and fatty acids) but not with leptin dose (Supplementary Fig. 6). In line with the above findings, in study 4, long-term leptin administration did not induce any significant changes in amino acids, ketone bodies, or lipoproteins compared to placebo (fatty acids were not assessed in this study, apart from free fatty acids (FFA)), which is indicated by the lack of distinct clusters in sPLS-DA and the lack of significantly different parameters in one-way ANOVA (Fig. 6e).

**Fig. 7:**
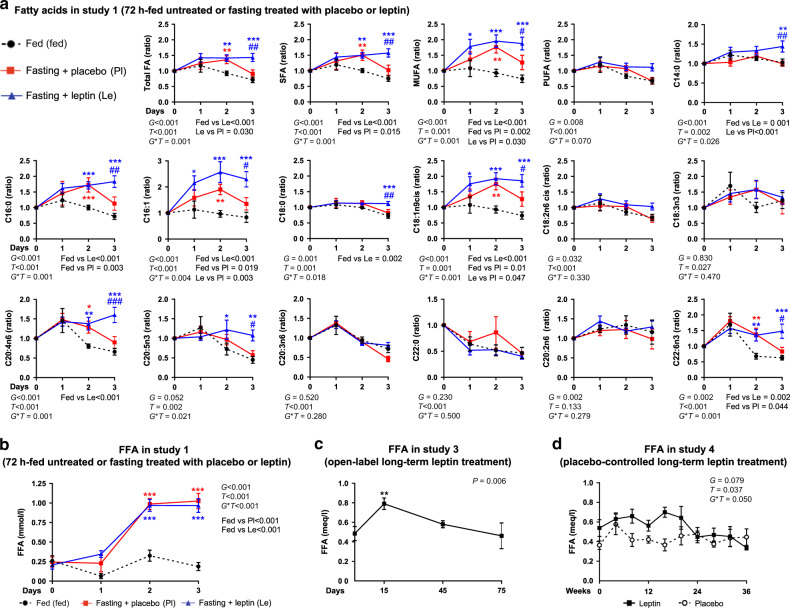
Fatty acid changes with short- and long-term leptin treatment. **a**, **b** Seventy-two hours fed state or fasting+leptin or fasting+placebo (study 1, *n* = 13). **a** Blood concentrations of fatty acid profile from start and till completion of the study as ratios of the baseline (0 day). GC/MS-EI was used to quantify fatty acid methyl esters in whole plasma. **b** Blood-free fatty acids (FFA) from start and till completion of the study; mixed model was performed (for FFA adjusted for baseline). *P* values of *G* (group: fed or fasting + placebo or fasting + leptin), *T* (time: days of study), and G*T interaction of mixed models are reported. In panels **a** and **b** by G*T < 0.05, post hoc Bonferroni test was performed between the estimated means of the three groups and between the three groups at each timepoint. One, two, or three asterisks indicate *P* < 0.05, <0.01, or <0.001 for fed vs fasting + placebo (red) and for fed vs fasting + leptin (blue). One, two, or three hash signs indicate *P* < 0.05, <0.01, or <0.001 for fasting + leptin vs fasting + placebo in the Bonferroni post hoc *t* test. **c** Open-label long-term leptin treatment in mildly hypoleptinemic women (study 3, *n* = 7). Blood FFA concentrations. *P* value of time effect (i.e., days of study) of mixed models adjusted for baseline is reported. Two asterisks indicate *P* < 0.01 for the specific timepoint vs 0 (baseline) in the Bonferroni post hoc *t* test (performed by *P* < 0.05). **d** Placebo-controlled long-term leptin treatment in mildly hypoleptinemic women (study 4, *n* = 19 (leptin = 10; placebo = 9)). *P* values of *G* (group: placebo or leptin), *T* (time: weeks of study), and G*T interaction of mixed models adjusted for baseline are reported. By G*T < 0.05, post hoc Bonferroni test was additionally performed between the two groups at each timepoint. One or two asterisks indicate *P* < 0.05, or <0.01 for leptin vs placebo for the specific timepoint. For Bonferroni post hoc tests, only significant results are reported. All *P* values are two-sided. Data are demonstrated as means ± SEMs. Exact *P* values. **a** Total FA (ratio) *G* = 0.008 × 10^−4^; *T* = 0.002 × 10^−1^; fed vs Le = 0.004 × 10^−1^; day 2 fed vs Pl = 0.008 & fed vs Le = 0.002; day 3 fed vs Le = 0.001 × 10^−1^ & Le vs Pl = 0.002. SFA (ratio) *G* = 0.002 × 10^−4^; *T* = 0.003 × 10^−2^; fed vs Le = 0.008 × 10^−5^; day 2 fed vs Pl = 0.003 & fed vs Le = 0.002; day 3 fed vs Le = 0.001 × 10^−1^ & Le vs Pl = 0.001. MUFA (ratio) *G* = 0.002 × 10^−6^; fed vs Le = 0.008 × 10^−7^; day 1 fed vs Le = 0.021; day 2 fed vs Pl = 0.001 & fed vs Le = 0.001 × 10^−1^; day 3 fed vs Le = 0.001 × 10^−1^ & Le vs Pl = 0.036. PUFA (ratio) *T* = 0.005 × 10^−3^. C14:0 (ratio) *G* = 0.009 × 10^−2^; Le vs Pl = 0.004 × 10^−1^; day 3 fed vs Le = 0.002 & Le vs Pl = 0.002. C16:0 (ratio) *G* = 0.004 × 10^−5^; *T* = 0.002 × 10^−2^; fed vs Le = 0.002 × 10^−5^; day 2 fed vs Pl = 0.008 × 10^−1^ & fed vs Le = 0.008 × 10^−1^; day 3 fed vs Le = 0.001 × 10^−1^ & Le vs Pl = 0.002. C16:1 (ratio) *G* = 0.002 × 10^−6^; *T* = 0.004 × 10^−2^; fed vs Le = 0.007 × 10^−7^; day 1 fed vs Pl = 0.012; day 2 fed vs Pl = 0.009 & fed vs Le = 0.001 × 10^−1^; day 3 fed vs Le = 0.001 × 10^−1^ & Le vs Pl = 0.014. C18:0 (ratio) day 3 fed vs Le = 0.001 × 10^−1^ & Le vs Pl = 0.008. C18:1n9cis (ratio) *G* = 0.001 × 10^−6^; fed vs Le = 0.006 × 10^−7^; day 1 fed vs Le = 0.022; day 2 fed vs Pl = 0.001 & fed vs Le = 0.001 × 10^−1^; day 3 fed vs Le = 0.001 × 10^−1^ & Le vs Pl = 0.039. C18:2n6cis (ratio) *T* = 0.001 × 10^−1^; C20:4n6 (ratio) *G* = 0.002 × 10^−1^; *T* = 0.002 × 10^−1^; fed vs Le = 0.001 × 10^−1^; day 2 fed vs Pl = 0.022 & fed vs Le = 0.004; day 3 fed vs Le = 0.001 × 10^−1^ & Le vs Pl = 0.008 × 10^−1^. C20:5n3 (ratio) day 2 fed vs Le = 0.025; day 3 fed vs Le = 0.004 & Le vs Pl = 0.038. C20:3n6 (ratio) *T* = 0.006 × 10^−12^; C22:0 (ratio) *T* = 0.005 × 10^−6^; C22:6n3 (ratio) *T* = 0.001 × 10^−3^; day 2 fed vs Pl = 0.004 & fed vs Le = 0.004; day 3 fed vs Le = 0.003 × 10^−1^ & Le vs Pl = 0.011. **b** *G* = 0.004 × 10^−11^; *T* = 0.005 × 10^−13^; G*T = 0.004 × 10^−3^; fed vs Le = 0.005 × 10^−10^; fed vs Pl = 0.003 × 10^−6^; day 2 fed vs Le 0.002 × 10^−7^ & fed vs Pl = 0.004 × 10^−5^; day 3 fed vs Le = 0.008 × 10^−9^ & fed vs Pl = 0.001 × 10^−10^. **c** Post hoc test at 15 days = 0.002. **d** Post hoc test at 8 weeks = 0.011 and at 16 weeks = 0.002.

Regarding the lipid profile, when we assessed explicitly the concentrations of FFA (Fig. 7b), as well as of triglycerides, phospholipids, and sphingomyelins bound to different size lipoproteins (Supplementary Data 1 and Supplementary Fig. 5c), no difference was observed between placebo and leptin in acute fasting of study 1. Interestingly, though component 3 consisting of ten parameters (Fig. 6d), mainly fatty acids, was able to discriminate 7 subjects (brown squares clustering together at lower half of the score plot—in the area of negative values of component 3) on day 3 of placebo treatment (Fig. 6c), thus indicating that the concentrations of these fatty acids are probably significantly different between placebo and leptin treatment on day 3. Indeed, the elevated concentrations of fatty acids (both free and bound to lipoproteins) are reduced partially to baseline level at the third day of placebo but not at the third day of leptin treatment (Fig. 7a). This shows that blocking hypoleptinemia not only does not prevent the fasting-induced changes in lipid metabolism, but it may slightly stimulate them.

In agreement with the mild stimulatory role of leptin on lipid catabolism during fasting, long-term leptin treatment in fed state led to a transient increase of the circulating levels of FFA. In study 3, FFA was increased at day 15 of treatment and returned to baseline later (Fig. 7c). In study 4, FFA, similar to study 3, was increased significantly in the leptin compared to the placebo group up to 20 weeks (*P* = 0.002 for treatment adjusted for baseline), but the significance of this change disappears when timepoints through 36 weeks are included (Fig. 7d). In examining relationships between FFA and the hypothalamic–pituitary–peripheral axes previously measured, we found a negative correlation of FFA with aldosterone for study 3 (*r* = −0.536 and *P* value = 0.047 adjusted for multiple timepoints/subjects), which was not confirmed in study 4 where both the changes in FFA and in aldosterone are milder (Supplementary Table 1). Finally, we did not find any association of FFA with thyroid-stimulating hormone (TSH), free triiodothyronine (T3), free thyroxine (T4), adrenocorticotropic hormone (ACTH), cortisol, renin, growth hormone-binding protein (GHBP), or insulin-like growth factor 1 (IGF-1) in the leptin group (Supplementary Table 1).

Altogether, leptin treatment does not induce a major shift from carbohydrate to lipid utilization, but it may affect fatty acid profile, either by maintaining very high fatty acid levels during short-term treatment in acute fasting or by transiently increasing FFA during long-term treatment. Importantly, these findings justify a more in-depth lipidomic analysis in the future, that will include lipid subgroups that were not assessed in our current study.

## Discussion

We investigated herein the effects of short- and long-term leptin treatment on the regulation of body weight and composition in lean normo- and mildly hypoleptinemic individuals in four clinical studies and observed important differences compared to the reported effects of leptin in animal models (ob/ob or lean) and in human studies with people in hypoleptinemic–lipodystrophic or obese–hyperleptinemic state. Our results support the hypothesis that leptin demonstrates differential effects on energy regulation, depending on the metabolic context and energy balance^25,36^, as reflected by leptin levels, with a progressive loss of function from conditions of energy and leptin deficiency to conditions of energy and leptin excess.

Regarding body weight, short-term leptin treatment does not further induce the weight loss observed during acute fasting in lean individuals. The lack of effect of leptin can be both due to the short duration of treatment and due to the abolishment of the effects of leptin on energy intake through the imposed complete fasting. Long-term leptin treatment in lean mildly hypoleptinemic women led to 4–4.5% of body weight loss (exclusively fat mass), which is far less compared to the weight loss observed in CLD^11^, modestly less than the weight loss observed in severe hypoleptinemia due to GL (~5.5%)^10^, and significantly more compared to the neutral weight effects observed in hyperleptinemic obesity. However, blood leptin levels at baseline, which ranged between 1.5 and 8 ng/ml, did not correlate with the % weight loss due to leptin treatment. This suggests that the response to leptin treatment in terms of weight loss may not depend linearly on the leptin blood concentrations, but may dependent on the metabolic context (i.e., energy status, presence of genetic mutations). Thus, defining strict thresholds in leptin blood concentrations as reliable predictors of weight loss with leptin treatment in obese populations may prove to be challenging and demands further studies in large populations with a wide range of leptin levels and different metabolic phenotypes.

Second, animal studies (mainly in ob/ob mice and in lean rodents) demonstrated that leptin administration prevents the expected reduction in energy expenditure due to low-energy intake^37^, potentially by acting on hypothalamic nuclei, upregulating SNS activity, and adrenal hormone secretion toward thermogenesis and increased HR and BP, and by increasing physical activity^38–44^. In humans, such effects are only modest, if any, as observed in CLD, GL, or PL^11,44–47^. In overweight/obese–hyperleptinemic people, leptin treatment does not affect energy expenditure^19,22,45^, apart from a reported improvement in non-resting energy expenditure with leptin replacement after stabilization to reduced body weight with diet, in a study of sequential study design^46,47^ and even if present this does not necessarily translate to better body weight sustainment^22,46,48^. We now show that leptin treatment does not increase resting energy expenditure, does not stimulate physical activity, and does not affect markers of SNS activity (HR, BP, cortisol, and catecholamine production) in the lean normoleptinemic and partial hypoleptinemic individuals of our studies, supporting the rather marginal, if any, effects of leptin on energy expenditure in humans.

Third, both stimulatory and inhibitory effects of leptin on lipolysis and lipid utilization have been reported based on the metabolic context (starvation or not), magnitude, and type of leptin deficiency (CLD, GL, and PL) and leptin dose. In rodents, starvation leads to hypoleptinemia and increased white adipose tissue (WAT) lipolysis via activation of the hypothalamic–pituitary–adrenal (HPA) axis^35,49^. Both WAT lipolysis and the activation of HPA are suppressed after physiologic leptin replacement, but stimulated after supraphysiologic leptin treatment^35^. In lean humans, a correlation between decreasing leptin levels and an increase in cortisol, FFA, and ketones during starvation was recently reported^34^, which suggested an anti-lipolytic role for leptin. In our study, administration of leptin in lean individuals does not attenuate the amino acid surge or the robust increase observed in circulating FFA and ketone bodies with fasting (even in very high leptin doses) and does not significantly affect cortisol or catecholamine levels. On the contrary, we observe higher concentrations of total fatty acids during the third day of leptin treatment compared to placebo, which supports a stimulatory, if any, and not an inhibitory effect of leptin on lipid catabolism. This is in agreement with observations in nonfasting conditions. Specifically, leptin stimulates lipid utilization in ob/ob mice^50^ and lipolysis in lean mice fed ad libitum possibly through activation of sympathetic neurons innervating adipocytes. In humans, similar to weight regulation, there is a progressive loss of the lipocatabolic effects of leptin from conditions of leptin deficiency to leptin excess. Consequently, in people with CLD, leptin replacement stimulates lipid catabolism (lipolysis and oxidation) as indicated by increases in ketone bodies, FFA, and acylcarnitines^51^. In people with GL or PL, leptin treatment has a modest effect on lipid catabolism, since it does not affect FFA and ketone body concentrations, but increases acylcarnitines and by-products of branched-chain amino acids and protein degradation^52^. Similarly, in our studies, leptin has a modest lipocatabolic effect in lean mildly hypoleptinemic women, as it is associated with a transient increase in FFA but no changes in ketone bodies or amino acid concentrations. Importantly, the increase in FFA was not associated with alterations in hypothalamic–pituitary function and specifically with thyroid hormones or IGF-1, which are known to have lipolytic effects^53,54^. It was only associated with the reduction observed in aldosterone levels in study 3, which was not verified in study 4, where dose adjustments were performed to prevent too much body weight loss. Given that aldosterone has rather lipolytic properties^55^, the transient, increased serum FFA may have an inhibitory effect on aldosterone secretion as a part of a compensatory mechanism. Furthermore, even though we see some small changes in FFA with leptin, these are not proportional to the changes in fat mass, suggesting that they may account for some minor but not all of leptin’s effects on body weight/fat mass that are probably due to the decreasing energy intake.

Regarding energy intake, in ob/ob mice and lean rodents, leptin replacement decreases caloric intake^9,37,56^. Similarly, in severe hypoleptinemic populations with CLD or GL, leptin decreases robust food intake by affecting hedonic and homeostatic nervous centers that control satiety and hunger feeling^31,57,58^. In lean women with mild acquired hypoleptinemia, leptin administration reduces salience, attention, and rewarding value of food^59^. In obese subjects after weight loss, which can be characterized as a condition of relative leptin deficiency, it affects brain activity and increases satiation^47,48^. In contrast, in obese–hyperleptinemic men, studied at their usual weight, leptin administration has minimal effects on appetite regulation^60^. In our studies, leptin administration in normoleptinemic lean subjects during short-term fasting partially prevents the increase of food intake at refeeding. Thus, it is plausible to expect similar effects on energy intake in partially hypoleptinemic individuals under long-term leptin treatment, considering the similar impacts on body weight in our longer-term trials. Indeed, the projection curve for the expected fat mass loss due to reduced energy intake almost overlaps the real curve of fat mass loss observed in study 3, where leptin dose was not adjusted based on body weight changes.

In summary, we present herein that one of the main metabolic effects of leptin in lean subjects is the regulation of energy intake, an effect that is saturable as leptin increases to within physiological levels at least during refeeding after food deprivation. This can be translated into weight loss, mainly due to fat mass loss, in the long term in subjects with chronic mild hypoleptinemia. Additionally, leptin treatment may lead to a transient increase in circulating FFA, without affecting energy expenditure and SNS activity. Although the effects of leptin on weight regulation, energy intake, and lipid catabolism are progressively lost with progression from conditions of energy and leptin deficiency to conditions of energy and leptin excess, the response to leptin treatment in terms of weight loss may not depend linearly on the leptin blood concentrations prior to treatment initiation. In the future, larger and longer studies of leptin administration to lean individuals in physiologic and supraphysiologic doses, as well as in the subset of obese patients with low endogenous leptin levels and/or obese subjects with induced hypoleptinemia, are needed to fully elucidate physiology and potential therapeutic utility of leptin in obesity^25,61^.

This study has some limitations. In short-term fasting studies, we measured RMR but not total or non-resting energy expenditure due to lack of metabolic chambers. Additionally, no weighted buffet meals to assess energy intake were performed longitudinally under long-term leptin replacement and this remains to be studied in detail in the future. Physical activity was calculated using daily self-report diaries as a surrogate of exercise-induced energy expenditure and this is a validated method. Our metabolite–lipid–lipoprotein analysis, although lege artis, did not include all circulating lipids or metabolites, and did not describe lipid subgroups and individual lipid species that should be the focus of more in-depth studies in the future. Additionally, whether the increase we observed in FFA in studies 3 and 4 is related to an upregulation of lipolysis, reduced lipogenesis or changes in re-esterification could not be addressed with certainty in the context of the current experimental setting. We also acknowledge that the sample size, especially in study 2, may have been small, resulting in increased type II error for some parameters. We have tried to address this by performing both combined for groups or doses/group analyses, as well as separate analyses for each group/dose. Finally, conclusions about SNS activity derive from catecholamines, HR and BP levels, and not from pharmacological blockade that may be able to detect very small differences or heart rate variability measures, which we have reported in the past^62^.

## Methods

We utilized data and specimens from our previous studies to perform new measurements and analyses^26–30^ (Supplementary Table 2). The primary outcomes of our analysis were (a) correlations of baseline leptin levels with % of weight change in all four clinical studies–interventions (Fig. 1 for study design), (b) differences in % weight changes between lean men, lean women, and obese in escalating leptin doses (study 2), and (c) weight, fat mass, and FFA changes in relation to leptin levels during long-term leptin treatment and after its termination (studies 3 and 4). The secondary outcomes were changes in energy expenditure (i.e., RMR and physical activity), energy intake, SNS activity (i.e., HR, BP, body temperature, and serum/urine catecholamines), and metabolic profile (i.e., lipoproteins, amino acids, fatty acids, and ketone bodies) in all four clinical studies–interventions (Fig. 1 for study design).

### Study approval

The human studies were approved by the Institutional Review Board of the General Clinical Research Center (GCRC) of the Beth Israel Deaconess Medical Center and were performed under an investigator-held IND. Written informed consent was obtained from all participants prior to inclusion in the study.

### Study 1: short-term mechanistic study

Eight healthy lean men (age = 23.3 ± 1.2 yr; BMI = 23.7 ± 0.6 kg/m^2^) and seven healthy lean women (age = 22.4 ± 1.2 yr; BMI = 21.7 ± 2.2 kg/m^2^) with regular menstrual cycles and not on oral contraceptives for at least 6 months were studied under three separate Clinical Research Center (CRC)-based conditions for 72 h: one under isocaloric fed-state conditions (normoleptinemia) and two during complete fasting-state conditions (induced hypoleptinemia) scheduled in a random order and in a double-blind fashion with administration of physiologic replacement of leptin doses (fasting + leptin) or placebo (fasting + placebo)^26,27^. The interval between admissions was at least 8 weeks to allow recovery of hematocrit, leptin levels, and body weight. Each subject completed three studies (i.e., fed, fasting + placebo, and fasting + leptin) with the following exceptions: two males withdrew before completing the fasting + leptin study and one female did not complete the fasting + placebo study. We excluded the two males from the analysis as their corresponding data were insufficient, but we included the female since she had completed 2/3 studies (fed and fasting + leptin), so that in total, findings from 13 subjects (6 males and 7 females) were analyzed. During each fed or fasting study, subjects were admitted to the CRC the evening before study day 0. The isocaloric fed state consisted of four standardized meals per day: breakfast (20% of daily calories) at 8:00, lunch (35% of daily calories) at 13:00, dinner (35% of daily calories) at 18:00, and a snack (10% of daily calories) at 22:00. During the fasting state, only a standardized volume of calorie-free fluids, electrolytes (NaCl (500 mg) and KCL (40 meq)), and vitamin supplements was allowed. Ad libitum feeding was allowed starting at 13:00 on the third study day and meals were weighed to obtain accurate measures of the calories ingested. Body composition (bioelectric impedance analysis; RJL Systems, Clinton Township, MI), RMR (DeltaTrac II Metabolic Monitor; SensorMedics), and morning vital signs (HR, BP) were assessed at the beginning and end of each study. Blood samples were obtained at 8:00–8:30 am on days 0, 1, 2, and 3. Urine collection was performed on day 2. The doses of leptin were 0.01 mg/kg given at 8 am and every 6 h on day 0 and 0.025 mg/kg at 8 am and every 6 h on days 1 and 2 for males and 0.02 mg/kg given at 8 am and every 6 h on day 0 and 0.05 mg/kg given at 8 am and every 6 h on days 1 and 2 for females (Amylin, Inc., San Diego, CA; previously known as r-metHuLeptin, provided by Amgen, Inc., Thousand Oaks, CA) administered subcutaneously (Fig. 1 for study design and Supplementary Fig. 7 for flow diagram). Males and females were administered a single dose of 0.025 mg/kg and 0.05 mg/kg, respectively, at 8 am on day 3. The results from these studies had previously been reported separately for men and women but are combined herein [ClinicalTrials.gov Study 1: NCT00140231].

### Study 2: short-term leptin dose escalation study

Five lean men (age = 22.2 ± 0.9 yr; BMI = 22.0 ± 0.5 kg/m^2^), five men with obesity (age = 23.4 ± 1.5 yr; BMI = 32.0 ± 1.0 kg/m^2^), and five lean women (age = 20.4 ± 0.7 yr; BMI = 21.9 ± 0.7 kg/m^2^) participated in 3 fed–normoleptinemic and 3 fasting-induced hypoleptinemic studies, which were conducted in the CRC, with leptin administration at three different doses (dose A = 0.01 mg/kg, dose B = 0.1 mg/kg, and dose C = 0.3 mg/kg) (protocol previously published^30^) (Fig. 1 for study design and Supplementary Fig. 7 for flow diagram). One normoleptinemic (fed) and one hypoleptinemic study (fasting) were performed at each of the three different doses of leptin, resulting in six visits in total. Leptin (metreleptin, supplied by Amgen, Inc., Thousand Oaks, CA) was administered once daily at 8:00 am subcutaneously. For males, fed studies were performed after the completion of all three fasting studies. For females, the first day of each fasting study was scheduled during the beginning of each follicular phase, and thus fed studies were conducted either in between or after the fasting studies.

The duration of each fasting-induced hypoleptinemic visit was 72 h. Subjects were admitted to the CRC the night before the first study day and received a standardized 748-kcal snack at 22:00. After that, subjects fasted until 22:00 of day 3 when they received a standardized 225-kcal snack. Only a standardized volume of calorie-free fluids, electrolytes (NaCl (500 mg) and KCL (40 meq)), and vitamin supplements were allowed from the beginning until the end of fasting on day 3. The interval between admissions was no less than 2 weeks.

The duration of each fed/normoleptinemic study was 24 h. Subjects were admitted to the CRC the night before the study day. On the study day, participants received an isocaloric diet with breakfast at 7:00 (20% of daily calories), lunch at 14:00 (35% of daily calories), dinner at 18:00 (35% of daily calories), and a snack at 22:00 (10% of daily calories), during which subjects received an isocaloric diet. Each admission was separated by 1–12 weeks. Eight subjects received the 0.01 mg/kg/day and the 0.1 mg/kg/day doses on consecutive days, since the 0.01 mg/kg/day dose was not expected to alter leptin levels 24 h later.

Vital signs, including HR, BP, body temperature, and respiratory rate, were measured at 7:00, 14:00, and 18:00–20:00 of each study day (e.g., 3 days for the hypoleptinemic state and 1 day for the normoleptinemic state). Body weight was measured on the morning of each study day, prior to blood sampling and prior to breakfast regarding the fed admissions, with the same scale in CRC and with subjects dressed in a standard hospital gown. Leptin was administered at 8:00 every morning. Leptin levels were measured at +30 min, +1 h, + 2 h, +3 h, +4 h, +5 h, +6 h, +8 h, +10 h, +12 h, +18 h, and +24 h after each dose (presented at the beginning and completion of treatment). Serum samples obtained in the early morning (prior to dose administration), noon (after leptin’s peak), and evening of each study day were used for renin, aldosterone, FFA, and lipoprotein/metabolite (nuclear magnetic resonance (NMR)-based metabolomics) measurements. For fatty acids with gas chromatography electron ionization mass spectrometry (GC/MS-EI), serum samples were examined on day 1 at 8:00 (before leptin administration), and on days 1, 2, and 3 at 14:00 (close to the serum peak of leptin). Finally, urine catecholamines were measured at baseline and on day 3 of each admission at the fasting state. Renin, aldosterone, and urine catecholamine measurements were not available in the fed state [ClinicalTrials.gov Study 2: NCT00140205].

### Study 3: long-term leptin replacement study

Eight lean women (age = 24.8 ± 5.4 years; body mass index (BMI) = 20.5 ± 2.0 kg/m^2^) with acquired hypoleptinemia due to hypothalamic amenorrhea (HA) secondary to strenuous exercise for at least 6 months were studied. All subjects were otherwise healthy, without active eating disorders, with stable weight (inclusion criteria: within ± 15% of ideal body weight for ≥6 months) and were not taking any medications, including estrogen, for at least 3 months. Finally, all participants had normal prolactin and thyrotropin levels, ratios of luteinizing hormone (LH) to follicle-stimulating hormone (FSH) of more than 1.5, and no signs of hirsutism or acne. Subjects received leptin (0.08 mg/kg/day, self-injected subcutaneously twice daily as 40% in the morning and 60% in the evening) initially for 2 months (protocol previously published^29^). Subjects who had not ovulated in the first 2 months continued with a third month of treatment at an increased dose of 0.2 mg/kg/day (with the same administration schedule as above). Ovulation was determined with one or more of the following: a 2 mm per day growth of the dominant follicle from its preovulatory size (≥18 mm in length), with subsequent collapse or internal echo appearance in the pelvic ultrasonography (performed weekly); serum or urinary LH surge; >4 ng per ml increase in progesterone levels. Blood samples were obtained weekly and body composition was determined with dual-energy X-ray absorptiometry (DEXA) every other week, starting 1 month before initiation of leptin treatment (baseline month, where measurements were performed at the beginning and end of the month). RMR was measured (DeltraTrac II Metabolic Monitor, SensorMedics) during the baseline month and after 15 days of leptin treatment. Morning vital signs (HR, BP, and temperature) were obtained in the morning during baseline month and after 15 days of leptin treatment. Daily exercise records were obtained. Physical activity was calculated as the weekly sum of the product of Mets (metabolic equivalent) *Duration (hours) for each activity type. Metabolic equivalent values used were according to the 2011 Compendium of Physical Activities (Supplementary Table 3)^63^. Even though eight females were initially enrolled, one subject withdrew after 1 month for reasons unrelated to the study, and thus, the results are derived from the remaining seven subjects (Fig. 1 for study design and Supplementary Fig. 7 for flow diagram).

### Study 4: confirmatory placebo-controlled study

Twenty females, between 18 and 35 years old with hypoleptinemia due to secondary HA for ≥6 months coincident with strenuous exercise and/or low body weight (within ± 15% of ideal body weight for ≥6 months at the time of screening), were studied. All subjects were otherwise healthy, without active eating disorders or other psychiatric disease and were not taking any medications that could affect hormone or bone mass measurements (i.e., glucocorticoids, antiseizure medications, thyroid hormones, or estrogens) for at least 3 months. None of the subjects had hyperprolactinemia, hypo- or hyperthyroidism, Cushing’s syndrome, congenital adrenal hyperplasia, or primary ovarian failure. Subjects were randomized with a 1:1 allocation to receive either metreleptin or placebo for 36 weeks^28^. Randomization tables were produced by the Harvard Catalyst biostatisticians with SAS and delivered directly to the Research Pharmacy for use such that study staff that recruited subjects (medical doctors, care providers) as well as the participants would remain blinded. Primary and secondary outcomes of the study were the difference between the placebo- and leptin-treated group for bone mineral content, bone markers, and bone mineral density, as well as reproductive outcomes from baseline to 36 weeks. Metreleptin was self-injected subcutaneously once daily at a dose of 0.08 mg/kg/day for 12 weeks, and subjects who had begun menstruating remained on this dose until the completion of the study. The dose for subjects who had not menstruated at week 12 was increased to 0.12 mg/kg/day. If a subject lost >5% of her baseline weight, the dose was reduced by 0.04 mg/kg. Fasting blood samples were collected every 4 weeks, along with fasting vital signs (HR, BP, and temperature) and body weight measurements. Body composition and RMR were measured every 12 weeks with DEXA and Sensormedics Vmax Encore equipment (VIASYS Respiratory Care Inc.), respectively. Physical activity was calculated as described for study 3. Among the 20 participants who were enrolled in the study, 11 were assigned randomly to receive metreleptin (age = 26.6 ± 1.4 years; BMI = 20.9 ± 0.6 kg/m^2^) and 9 to receive placebo (age = 25.4 ± 1.2 years; BMI = 19.8 ± 0.7 kg/m^2^). One participant in the metreleptin-treated group withdrew from the study because she developed injection-site reactions soon after the baseline visit, leaving 10 in the metreleptin group and 9 in the placebo group (Fig. 1 for study design and Supplementary Fig. 7 for flow diagram) [ClinicalTrials.gov Study 4: NCT00130117].

### Biochemical analysis

FFA (intra-assay variability: 1.5%, sensitivity: 0.01–4.00 mEq/L NEFA) was measured using commercially available enzymatic colorimetric assay from Fujifilm Wako Diagnostics U.S.A Corporation (Mountain View, CA, USA). Levels were measured with an automated immunoassay system (Immulite 1000, Siemens, Deerfield, IL). All samples were run in duplicates within the same run for a given subject and were repeated if the coefficient of variation for any sample was N15%. Aldosterone (intra-assay CV 1.4–3.4%, inter-assay CV 9.5–12.1%, and sensitivity: 22.4 pg/mL) and renin (intra-assay CV 1.7–5.3%, inter-assay CV 4.0–5.5%, and sensitivity: 14.8 pg/mL) were measured using commercially available immunoassays (R&D Systems, Inc., Minneapolis, MN, USA). Levels were measured with an automated immunoassay system (Immulite 1000, Siemens, Deerfield, IL). Similar to the FFA analysis, all samples were run in duplicates within the same run for a given subject and were repeated if coefficient of variation for any sample was >15%.

### NMR-based metabolomics

High-throughput proton NMR metabolomics (Nightingale Health Ltd, Helsinki, Finland) was used to quantify circulating metabolites and lipids within lipoprotein particles. This is a targeted metabolomics approach where all metabolic measures are of known identity and therefore are in level 1 identification level according to Summer et al.^64^. The method leads to simultaneous quantification of lipoprotein subclasses with lipid concentrations, fatty acids, amino acids, ketone bodies, and metabolites related to gluconeogenesis (Nightingale Health biomarker quantification library 2016). Details of the experimentation and proton NMR spectrometer characteristics have been described previously^65–67^. In brief, serum samples are stored in a freezer at −80 °C. Before preparation, frozen samples are slowly thawed at +4 °C overnight and then mixed gently and centrifuged at 3400×*g* to remove possible precipitate. Aliquots of each sample (100 μl) are added to 100 μl of sodium phosphate buffer (75 mM Na_2_HPO_4_ in 80%/20% H_2_O/D_2_O, pH 7.4, including also 0.08% sodium 3-(trimethylsilyl)propionate-2,2,3,3-d4 and 0.04% sodium azide) automatically with a Gilson Liquid Handler 215 to 3-mm outer-diameter SampleJet NMR tubes. The resulting solution is then mixed by aspirating three times. The prepared samples are stored in 96-tube racks that are inserted into one of the five well-plate positions in the SampleJet^TM^ (Bruker BioSpin GmbH, Germany) sample changer. The latter is placed on top of the superconducting magnet inside which the NMR probehead is located. The sample changer includes a cooling unit, which maintains the temperature of samples waiting to be measured at +6 °C, and a preheating unit, which warms up the sample just before measurement. The sample is then kept idle inside the NMR probehead to achieve temperature stabilization at 36.95 °C. Thus, the measurement temperature is constant. Two NMR spectra are acquired from each serum sample using a Bruker AVANCE III spectrometer operating at 500.36 MHz. The first spectrum includes overlapping resonances arising mainly from different lipid molecules in various lipoprotein particles. The second spectrum, acquired with spectrometer settings using a T2-relaxation-filtered pulse sequence to suppress most of the broad macromolecule and lipoprotein lipid signals, leading to enhanced detection of rapidly tumbling smaller solutes. Representative spectra are illustrated in reviews of the Nightingale NMR metabolomics method^65^. Data processing includes the Fourier transformations to NMR spectra, automated phase correction, overall signal check for missing/extra peaks, background control, baseline removal, and spectral area-specific signal alignments, and comparisons with the spectra of the two quality control samples. The NMR metabolomics (Nightingale Health Ltd., Helsinki, Finland) method has also been used and described in refs. ^68–73^. The effects of these experimental aspects on the metabolic biomarker concentrations are best reflected in the coefficients of variation (CV) for the measurements, and representative CVs have been published previously in the supplement of refs. ^73,74^ in which mean CV (%) was 4.5% and 5.0%, respectively. The relation of quantified biomarker data to annotated NMR spectral data has recently been discussed extensively in ref. ^72^ and additional spectral data have been made publicly available in (https://www.ebi.ac.uk/metabolights/MTBLS974). The lipoprotein lipid measures were quantified using gel permeation high-performance liquid chromatography (GP HPLC) as calibration reference, as described previously^65,66,75^. The GP HPLC assay captures the cholesterol, triglyceride, and phospholipid levels in lipoprotein subclasses, which in turn are accurately reproduced in the Nightingale NMR platform in a high-throughput manner (https://pubmed.ncbi.nlm.nih.gov/32359769/). The quantified metabolite measures have also been compared with alternatively analytical methods for measuring the same metabolites, including two commercial mass-spectrometric platforms, showing good consistency^76^. Numerous published large epidemiological studies have used the Nightingale Health platform (see https://nightingalehealth.com/publications for an overview) and the platform is currently being used to measure all 500,000 samples from the UK Biobank^77^. For this study, and all prior publications using the Nightingale platform, we used the quantified biomarker measures in absolute concentration units or ratios provided directly from the commercial metabolomics platform, and no raw spectral data were used in epidemiological analyses.

### Quantification of serum fatty acids

Fatty acid methyl esters (FAMEs) from whole plasma were determined as follows. In a chloroform-resistant Eppendorf, 30 µL of plasma were spiked with 65 µL of Internal standard (ISTD) nonadecanoic acid (C19:0) (Merck) 100 µg/mL solution (6.5 µg). Spiked plasma was extracted with chloroform–methanol (2:1, v/v). Organic phase was transferred into a screw-cap test tube and evaporated to dryness under N2 at 37 °C. Plasma fatty acids were hydrolized and methylated following an adaptation of the method described by Agren et al.^78^. Briefly, 100 µL of n-toluene and 500 µL of boron trifluoride–methanol solution (14%) were added to the tube, which was capped and placed into a block heather (100 °C) for 60 min. After cooling, 500 µL of distilled water and 500 µL of n-hexane were added. After shaking for 1 min, the tubes were centrifuged for 5 min at 2200 × *g* at room temperature to separate the layers. The hexane layer was placed into a test tube and evaporated to dryness under N_2_ at 30 °C. The extracts were reconstituted with 100 µL of n-hexane and transferred into an automatic injector vial equipped with a glass insert of 300 µL.

FAMEs were analyzed by GC/MS-EI using an Agilent 6890 N GC equipped with an Agilent 7683 autosampler, and an Agilent 5973 N mass spectrometry detector. FAMES was separated with a J&W DB-FastFAME capillary column (30 m × 0.2 mm × 0.25-μm film thickness) (Agilent). The injector temperature was set at 250 °C, and 1-μL injections were made (split ratio 25:1). GC was run using an optimized temperature program as follows: the temperature program started at 50 °C, held for 0.5 min, increased to 194 °C at a rate of 25 °C/min, held for 1 min, and increased to 245 °C at the rate of 5 °C/min, held for 3 min. Helium was used as a carrier gas (14 psi, constant pressure mode). FAMEs were detected using selected ion monitoring (SIM) mode. Several m/z ions common to saturated, monounsaturated, and polyunsaturated FAMES were monitored (see detailed information in Supplementary Table 4). All data were quantified by integrating the area under the curve of each metabolite using MassHunter Quant (Agilent Technologies). Nine mixtures of FAME external calibration standards were prepared by dilution in hexane of certified FAME reference material mix (Supelco 37 Component FAME Mix, Merck) and kept at −20 °C until analysis. About 30 µL of each mixture was added to a tube, was spiked with 650 µL of ISTD C19:0 methyl ester 100 µg/mL solution (6.5 µg), evaporated to dryness under N_2_ at 30 °C, reconstituted with 100 µL of hexane, and transferred into an automatic injector vial equipped with a glass insert of 300 µL. The equivalents of C19:0 added to the samples, as free fatty acid ISTD, were the same as the amount of C19:0–methyl ester added to the external calibrators. The concentration of FAMES in the samples was calculated by linear regression of the peak area ratio relative to that of the internal standard.

### Statistics and reproducibility

Statistical analysis was performed with SPSS v19.0 (SPSS, Inc., Chicago, IL) for Windows, with GraphPad prism 7 (GraphPad Software Inc., La Jolla, CA), and with MetaboanalystR^79^. The results are presented in figures and tables as mean ± SEM. In study 3 (*n* = 7), individual data points are shown. Mixed model adjusted for baseline was used for all parameters (unless otherwise specified). In fatty acids deriving from GC/MS-EI, where values were normalized to baseline (thus, ratios were created), mixed model without adjustment for baseline was used. Compound symmetry was selected as repeated covariance type. Factors were time (corresponded to days or hours of fasting for study 1 and study 2, days of treatment for study 3, and weeks of treatment for study 4) and group (fed, fasting treated with placebo, and fasting treated with leptin for study 1; physiologic (0.01 mg/kg), supraphysiologic (0.1 mg/kg), pharmacologic (0.3 mg/kg) for study 2, and leptin or placebo for study 4). In study 2, two additional analyses were performed that included as factors time (corresponded to hours of fasting or to hours after leptin administration in fed state) and group (lean men, lean women, and obese men) for each leptin dose separately. When only the group factor- existed p value was calculated with unpaired *t* test (e.g., Fig. 2a leptin at baseline and % weight change), with one-way ANOVA (Fig. 2b leptin at baseline and % weight change), and with repeated measure ANOVA (Fig. 3a food intake and Fig. 5a urine catecholamines). When only the time factor existed and only two timepoints were available for analysis, paired *t* test was used (study 3: Fig. 3e RMR and temperature, Fig. 4c and Supplementary Fig. 4b). Post hoc Bonferroni corrections were performed between the total means of the groups, as well as between the means of the groups in the individual timepoints when the time*group *P* value < 0.05. When only the group factor existed, post hoc Bonferroni corrections were performed when *P* < 0.05. When only the time factor existed, post hoc Bonferroni’s test comparing the timepoint vs baseline was performed when *P* < 0.05. Curve estimation was performed in SPSS to choose the best fit and ANOVA to calculate the corresponding *P* values for the logarithmic curve to show relationships between variables. One outlier according to the ROUT method (GraphPad) with a Q = 0.1% was detected and removed from the urine cortisol measurements in study 1 and from the FFA measurements of study 4. Pearson’s or Spearman’s (when data were not normally distributed) correlation tests were used for correlations between variables. For the correlations, an additional analysis by using repeated measure correlation (rmcorr package for R) was performed, in order to adjust for the fact that some subjects have contributed in the correlation more than one point. This was the case for (a) study 2, correlation of baseline leptin levels with % of weight loss (Supplementary Fig. 1a, adjustment was performed since each subject has contributed three points corresponding to three different leptin doses), (b) study 1, correlation of food intake to baseline leptin levels in an ad libitum meal intake after fasting (Supplementary Fig. 1e, adjustment was performed for fasting since each subject has contributed two points, corresponding to leptin or placebo treatment; values were logarithmically transformed since they were not linear), and (c) study 3 and Study 4, correlations of hormones with FFA (Supplementary Table 1, adjustment was performed, since each subject has multiple FFA and hormonal measurements in different timepoints).

For study 1, measurements from NMR and GC/MS-EI were analyzed together both for all three admissions (Fig. 6a—fed, fasting + leptin, fasting + placebo) as well as only for the two fasting admissions (Fig. 6c, d—fasting + leptin, fasting + placebo). For study 2 and study 4, measurements from NMR (but not from GC/MS-EI) were available and analyzed (Supplementary Fig. 6 for study 2 and Fig. 6e for study 4). Three parameters (i.e., pyruvate, C18:1trans, C24:0) and five timepoints (i.e., in day 1 two subjects treated with leptin and one with placebo, in day 2 one treated with placebo, and in day 3 one treated with leptin) had more than 50% missing values (due to low or no detection, lack of quantification due to sample irregularities, or rejection by automatic quality controls) and were excluded from the analysis in study 1. Similarly, four parameters (pyruvate, glycerol, phenylalanine, and C18:1trans) in study 2 and one parameter (pyruvate) in study 4 had more than 50% missing values and were excluded. In all other cases, the missing values (5,4% of the whole data set) were replaced by half of the minimum value in the original data set to create a processed data set. Subsequently, the data from the processed data set were mean-centered and divided by the standard deviation of each variable to create a normalized–scaled data set. The means ± SEM of the processed data set for study 1 for all three admissions is provided in Supplementary Data 1, as well as for selected (most important) parameters of study 1 and study 2 in Supplementary Fig. 5 and Supplementary Fig. 6b–d, respectively. In Fig. 6a, the normalized–scaled data set for all three admissions was used to perform a one-way ANOVA with an adjusted FDR to the number of measurements (*0.05/n)*, i.e., 2.15 × 10^−4^. This identified 68 significantly different parameters between the three admissions, which were then used to create the heatmap demonstrated in Fig. 6b. In study 1, 2, and 4, sparse partial least-squares discriminant analysis (sPLS-DA) was performed with a setup of five components, each consisting of a maximum of 10 variables and with a fivefold CV validation. Similarly, the normalized–scaled data set for the two admissions (fasting + placebo vs fasting + leptin) was used to perform the sPLS-DA analysis and present the score plot and parameters involved in components 1 and 3 in Fig. 6c, d (study 1). Similarly, the normalized–scaled data set was used to perform the one-way ANOVA, the sPLS-DA, and present the score plot in study 4 (demonstrated in Fig. 6e). Finally, the normalized–scaled data set that included the measurements at start and completion of each admission was used to perform the sPLS-DA with score plot and component 2 in Study 2 (demonstrated in Supplementary Fig. 6a).

In study 1, for the five timepoints excluded from combined NMR and GC/MS-EI sPLS-DA and one-way ANOVA analysis in Fig. 6 due to >50% missing values, FAME values were though available. Thus, an additional analysis of FAMEs was performed that included these 5 timepoints. In this analysis, additionally the FAME ratio was calculated, to adjust for baseline differences due to lipid extraction—lipid volume loaded as well as due to expected physiological variability between three admissions performed in different timepoints. Missing baseline measurement of an admission (i.e., fed or fasting + leptin or fasting + placebo) was replaced with the average of the baseline values of the other two admissions of the same subject. Ratios were then calculated and analyzed with mixed models without adjustment for baseline and are presented in Fig. 7.

Regarding study reproducibility, all studies have been performed in the past. Independent repeat of any part of the experiment—studies were neither in our aims nor possible due to restrictions in the administration of leptin in humans with the exception of people with congenital leptin deficiency or generalized lipodystrophy where an FDA approval exists. Thus, we have (a) used existing data from older measurements (see Supplementary Table 2), (b) we have performed new measurements in already-collected and appropriately stored samples from the above studies. New hormonal measurements (i.e., renin and aldosterone) and FFA measurements of the samples were performed in duplicate and were repeated if coefficient of variation for any sample was >15%. The value deriving from the repetition was considered the valid one if the coefficient of variation was <15%. The metabolite–lipoprotein–fatty acid measurements were performed by state-of-the-art methods described above, with de-identified samples and blinded personnel regarding the different groups.

### Additional resources

Clinical trial registry numbers are the following: study 1: NCT00140231; study 2: NCT00140205; study 4: NCT00130117 available in ClinicalTrials.gov. Flow diagrams of all four studies are available in the Supplementary Material (Supplementary Fig. 7).

### Reporting summary

Further information on research design is available in the Nature Research Reporting Summary linked to this article.

## Supplementary information

Supplementary Information

Peer Review File

Description of Additional Supplementary Files

Supplementary Data 1

Supplementary Data 2

Reporting Summary

## Data Availability

Any other data that support the findings of this study are available from the corresponding author upon reasonable request. A list of all the identified metabolites along with relevant identifying information is available in Supplementary Data 2. Source data are provided with this paper.

## Source data

Source Data
