# Supplementary material for: Leptin alters energy intake and fat mass but not energy expenditure in lean subjects
Source: Nat Commun. 2020 Oct 13;11:5145. doi: 10.1038/s41467-020-18885-9 (PMC7553922; doi:10.1038/s41467-020-18885-9)
Supplement: Supplementary file 1 — Supplementary Information [file 41467_2020_18885_MOESM1_ESM.pdf]

Supplementary Table 1: Correlations of FFA with hormones in Study 3 (open-label long-term leptin treatment) and 4 (placebo-controlled long-term leptin treatment)

Hormones	Study 3 (n=7)		Study 4 (Leptin Group [n=10])	
	FFA (meq/l)		FFA (meq/l)	
	R	P	R	P
**TSH (µIU/ml)**	0.348	0.221	0.123	0.300
**FT3 (pg/ml)**	-0.391	0.166	0.106	0.371
**FT4 (ng/dl)**	-0.428	0.125	-0.078	0.509
**ACTH (pg/ml|)**	-0.298	0.402	ND	ND
**Cortisol (µg/dl)**	ND	ND	0.092	0.437
**Aldosterone (pg/ml)**	-0.536	0.047	-0.027	0.825
**Renin (pg/ml)**	-0.164	0.575	-0.026	0.832
**GHBP (pmol/l)**	0.071	0.807	ND	ND
**IGF-1 (ng/ml)**	0.438	0.116	-0.160	0.185

Repeated measures correlations were performed with “rmcorr” package in R studio. P-values reported are two-sided.
R: correlation coefficient; P: p-value; TSH: thyroid-stimulating hormone; FT3: free triiodothyronine; FT4: free thyroxine; ACTH: adrenocorticotropic hormone; GHBP: growth hormone-binding protein; IGF-1: insulin-like growth factor 1; ND: Data not available

Supplementary Table 2: Documentation of published, unpublished and existing raw data analyzed differently to answer new questions, presented in all figures.

		Published	Existing raw data from prior studies are analyzed differently herein to answer a new question	Unpublished
Figure 2	a		In study 1, leptin and weight have been published for males[1] and females[2] separately and as a combined analysis[3-5] to show no effect of short-term leptin treatment on weight loss during 72-hour fasting, but here we analyzed all the original data de novo in order to answer a novel question i.e. whether baseline leptin levels (prior to treatment initiation) correlate with % of weight change after 72-hour fasting treated with placebo or leptin at replacement doses.	
	b		In study 2, leptin and weight have been published to demonstrate changes in these variables during 72-hour fasting separately for each group*dose combination (i.e.dose A in lean men, dose B in lean men, C in lean men, A in lean women, B in lean women etc.)[6], but here we analyzed the original raw data de novo in order to answer following novel questions: a) Do baseline leptin levels (prior to treatment initiation) correlate with % of weight change observed after 72-hour of fasting treated with three escalating leptin doses, when lean men, lean women and obese men were studied both separately as well as together? b) Are the % weight changes observed under the three escalating leptin doses significantly different between lean men, lean women and obese men?	
	c	Mean leptin, weight and fat mass have been published for studies 3 and 4[7,8] but data are analyzed again and are presented here as change from baseline (Δ) in order to answer following questions: Are the absolute (delta) changes in leptin levels associated with the changes observed in body weight and fat mass during leptin treatment? Do these changes reverse after treatment completion?	Mean leptin and weight have been published for studies 3 and 4[7,8] (women with HA) to show weight-reducing effects of long-term leptin replacement, but here we analyzed the original raw data in order to answer following novel question: Do baseline leptin levels (prior to treatment initiation) correlate with % of weight change observed after 8 weeks of treatment with leptin? For this question we have combined the raw data from study 3 and study 4 in our analysis.	

**Figure 3**	**a**		In study 1, food intake has been published for males[1], but neither for females nor as combined analysis. Here we present for the first time food intake as a combined analysis of males and females, and explore whether leptin levels prior to meal correlate with caloric intake after 72-hour fasting treated with placebo or leptin at replacement doses.	
	**b**			Expected fat mass loss in studies 3 and 4 based on leptin-induced caloric deficit demonstrated in study 1.
	**c**		In study 1, RMR has been published separately for males[1] and females[2,9], but not as a combined analysis. Here we present for the first time RMR as a combined analysis of data from males and females to increase sample size and power.	
	**d**			Temperature and respiratory rate in study 2
	**e**	RMR in study 3[8]		Temperature, exercise in study 3
	**f**			RMR, temperature, exercise in study 4
**Figure 4**	**a**		In study 1, HR and BP have been published for females[9], but neither for males nor as a combined analysis. Here we present for the first time HR and BP as a combined analysis of data from males and females to increase sample size and power.	
	**b**			HR, SBP, DBP and MBP in study 2
	**c**			HR, SBP, DBP and MPB in study 3
	**d**			HR, SBP, DBP and MBP in study 4
**Figure 5**	**a**		In study 1, aldosterone, cortisol and urine catecholamines have been published separately for males[1] and females[2,9], but not as a combined analysis. Here we present for the first time these variables as a combined analysis of males and females to increase sample size and power.	
	**b**			Aldosterone, renin and urine catecholamines in study 2

	c			Aldosterone and renin in study 3
	d			Aldosterone, renin, free cortisol and urine catecholamines in study 4
Figure 6	a-d			Metabolite profile in study 1
	e			Metabolite profile in study 4
Figure 7	a			Fatty acids in study 1
	b		In study 1, FFA have been published separately for males[1] and females[2], but not as a combined analysis. Here we present for the first time FFA as a combined analysis of data from males and females to increase sample size and power.	
	c			FFA in study 3
	d			FFA in study 4
Supplementary Figure 1	a		Mean leptin and weight have been published for study 2, but a potential correlation between these variables has not been investigated previously[6]. Here we present for the first time an analysis to explore whether leptin levels at baseline (before treatment initiation) correlate with % weight change after 72-hour fasting treated with escalating doses of leptin separately in each group (i.e. lean men, obese men, lean women) after adjusting for intra-individual variability.	
	b		Mean weight has been published for study 2, to demonstrate change with fasting separately for each group (i.e. lean men, obese men, lean women)*dose (i.e. 0.01, 0.1, 0.3mg/kg) combination, but not as a within group analysis for time and dose effect[6]. Here we present for the first time a within group analysis to explore potential leptin-dose dependent effects on %weight change after 72-hour fasting separately in lean men, obese men and lean women.	
	c	Lean mass in study 3 presented as % change from baseline[8]		

	**d**	Lean mass in study 4 presented as % change from baseline[10]		
	**e**		In study 1, food intake has been published for males[1], but neither for females nor as combined analysis. Here we applied a combined analysis of males and females to explore whether leptin levels prior to meal correlate with caloric intake after 72-hour fasting treated with placebo or leptin at replacement doses, after adjusting for intra-individual variability.	
**Supplementary Figure 2**	**a**		In study 2, leptin and weight have been published for the three fasting admissions separately for each dose (i.e. 0.01, 0.1, 0.3 mg/kg)*group (i.e. lean men, obese men, lean women) combination, but not as a within dose analysis for time and group effect[6]. Here we present for the first time a within dose A (i.e. 0.01mg/kg/day) analysis to explore physiological leptin levels' effects on weight change during 72-hour fasting in lean men vs obese men vs lean women.	Respiratory rate, temperature, FFA, aldosterone, renin, urine catecholamines, HR, SBP and DBP in study 2. A within dose A (i.e. 0.01mg/kg/day) analysis was performed to explore physiological leptin levels' effects on these variables during 72-hour fasting in lean men vs obese men vs lean women.
	**b**		In study 2, leptin and weight have been published for the three fasting admissions separately for each dose(i.e. 0.01, 0.1, 0.3 mg/kg)*group (i.e. lean men, obese men, lean women) combination, but not as a within dose analysis for time and group effect[6]. Here we present for the first time a within dose B (i.e. 0.1mg/kg/day) analysis to explore supraphysiological leptin levels' effects on weight change during 72-hour fasting in lean men vs obese men vs lean women.	Respiratory rate, temperature, FFA, aldosterone, renin, urine catecholamines, HR, SBP and DBP in study 2. A within dose B (i.e. 0.1mg/kg/day) analysis was performed to explore supraphysiological leptin levels' effects on these variables during 72-hour fasting in lean men vs obese men vs lean women.
	**c**		In study 2, leptin and weight have been published for the three fasting admissions separately for each dose(i.e. 0.01, 0.1, 0.3 mg/kg)*group (i.e. lean men, obese men, lean women) combination, but not as a within dose analysis for time and group effect[6]. Here we present for the first time a within dose C (i.e. 0.3mg/kg/day) analysis to explore pharmacological leptin levels' effects on weight change during 72-hour fasting in lean men vs obese men vs lean women.	Respiratory rate, temperature, FFA, aldosterone, renin, urine catecholamines, HR, SBP and DBP in study 2. A within dose C (i.e. 0.3mg/kg/day) analysis was performed to explore pharmacological leptin levels' effects on weight change during 72-hour fasting in lean men vs obese men vs lean women.

Supplementary Figure 3	a		In study 2, leptin has been published for the three fed admissions separately for each dose (i.e. 0.01, 0.1, 0.3 mg/kg)*group (i.e. lean men, obese men, lean women) combination, but not as a within dose analysis for time and group effect[6]. Here we present for the first time a within dose A (i.e. 0.01mg/kg/day) analysis to explore physiological leptin levels' effects on weight change during fed state in lean men vs obese men vs lean women.	Respiratory rate, temperature, FFA, HR, SBP and DBP in study 2. A within dose A (i.e. 0.01mg/kg/day) analysis was performed to explore physiological leptin levels' effects on these variables during fed state in lean men vs obese men vs lean women.
	b		In study 2, leptin has been published for the three fed admissions separately for each dose (i.e. 0.01, 0.1, 0.3 mg/kg)*group (i.e. lean men, obese men, lean women) combination, but not as a within dose analysis for time and group effect[6]. Here we present for the first time a within dose B (i.e. 0.1mg/kg/day) analysis to explore supraphysiological leptin levels' effects on weight change during fed state in lean men vs obese men vs lean women.	Respiratory rate, temperature, FFA, HR, SBP and DBP in study 2. A within dose B (i.e. 0.1mg/kg/day) analysis was performed to explore supraphysiological leptin levels' effects on these variables during fed state in lean men vs obese men vs lean women.
	c		In study 2, leptin has been published for the three fed admissions separately for each dose (i.e. 0.01, 0.1, 0.3 mg/kg)*group (i.e. lean men, obese men, lean women) combination, but not as a within dose analysis for time and group effect[6]. Here we present for the first time a within dose C (i.e. 0.3mg/kg/day) analysis to explore pharmacological leptin levels' effects on weight change during fed state in lean men vs obese men vs lean women.	Respiratory rate, temperature, FFA, HR, SBP and DBP in study 2. A within dose C (i.e. 0.3mg/kg/day) analysis was performed to explore pharmacological leptin levels' effects on these variables during fed state in lean men vs obese men vs lean women.
Supplementary Figure 4	a		In study 1, RQ has been published separately for males[1] and females[2] and macronutrient utilization has been published only for males[1], but not as a combined analysis. Here we present for the first time RQ and macronutrient utilization as a combined analysis of males and females to increase sample size and power.	
	b			RQ and macronutrient utilization in study 3
	c			Macronutrient utilization in study 4
Supplementary Figure 5	a			Ketone bodies in study 1
	b			Amino acids in study 1
	c			Lipoproteins in study 1

**Supplementary Figure 6**	**a**			Metabolite profile in study 2
	**b**			Lipoproteins in study 2
	**c**			Ketone bodies and amino acids in study 2
	**d**			Fatty acids in study 2

FFA, free fatty acids; RMR, resting metabolic rate; HR: heart rate; SBP, systolic blood pressure; DBP. diastolic blood pressure; MBP, mean blood pressure; RQ, respiratory quotient.

Supplemental Table 3: Metabolic equivalents (MetS) used to calculate physical activity in Study 3 (open-label long-term leptin treatment) and 4 (placebo-controlled long-term leptin treatment)

Type of exercise as specified by subject	MetS	Category
**Elliptical**	**5**	conditioning exercise
**Power Yoga**	**4**	conditioning exercise
**Kick Boxing**	**10.3**	sports
**Walking***	**4.15**	
walking, less than 2.0 mph, level, strolling, very slow	*2*	walking
walking, 2.0 mph, level, slow pace, firm surface	*2.8*	walking
walking for pleasure (Taylor Code 010)	*3.5*	walking
walking from house to car or bus, from car or bus to go to places, from car or bus to and from the worksite	*2.5*	walking
walking to neighbor's house or family's house for social reasons	*2.5*	walking
walking the dog	*3*	walking
walking, 2.5 mph, level, firm surface	*3*	walking
walking, 2.5 mph, downhill	*3.3*	walking
walking, 2.8 to 3.2 mph, level, moderate pace, firm surface	*3.5*	walking
walking, 3.5 mph, level, brisk, firm surface, walking for exercise	*4.3*	walking
walking, 2.9 to 3.5 mph, uphill, 1 to 5% grade	*5.3*	walking
walking, 2.9 to 3.5 mph, uphill, 6% to 15% grade	*8*	walking
walking, 4.0 mph, level, firm surface, very brisk pace	*5*	walking
walking, 4.5 mph, level, firm surface, very, very brisk	*7*	walking
walking, 5.0 mph, level, firm surface	*8.3*	walking
walking, 5.0 mph, uphill, 3% grade	*9.8*	walking
walking, for pleasure, work break	*3.5*	walking
walking, grass track	*4.8*	walking
walking, normal pace, plowed field or sand	*4.5*	walking
walking, to work or class (Taylor Code 015)	*4*	walking
walking, to and from an outhouse	*2.5*	walking
walking, for exercise, 3.5 to 4 mph, with ski poles, Nordic walking, level, moderate pace	*4.8*	walking
walking, for exercise, 5.0 mph, with ski poles, Nordic walking, level, fast pace	*9.5*	walking
walking, for exercise, with ski poles, Nordic walking, uphill	*6.8*	walking
**Jogging***	**6.5**	
jog/walk combination (jogging component of less than 10 minutes) (Taylor Code 180)	*6*	running

jogging, general	*7*	running
jogging, in place	*8*	running
jogging, on a mini-tramp	*4.5*	running
**Yoga***	**2.5**	
yoga, Hatha	*2.5*	conditioning exercise
yoga, Nadisodhana	*2*	conditioning exercise
yoga, Surya Namaskar	*3.3*	conditioning exercise
**Slide***	**11**	conditioning exercise
**Running***	**11.25**	
running, 4 mph (13 min/mile)	*6*	running
running, 5 mph (12 min/mile)	*8.3*	running
running, 5.2 mph (11.5 min/mile)	*9*	running
running, 6 mph (10 min/mile)	*9.8*	running
running, 6.7 mph (9 min/mile)	*10.5*	running
running, 7 mph (8.5 min/mile)	*11*	running
running, 7.5 mph (8 min/mile)	*11.5*	running
running, 8 mph (7.5 min/mile)	*11.8*	running
running, 8.6 mph (7 min/mile)	*12.3*	running
running, 9 mph (6.5 min/mile)	*12.8*	running
running, 10 mph (6 min/mile)	*14.5*	running
running, 11 mph (5.5 min/mile)	*16*	running
running, 12 mph (5 min/mile)	*19.0*	running
running, 13 mph (4.6 min/mile)	*19.8*	running
running, 14 mph (4.3 min/mile)	*23*	running
running, cross country	*9*	running
running, (Taylor code 200)	*8*	running
running, stairs, up	*15*	running
running, on a track, team practice	*10*	running
running, training, pushing a wheelchair or baby carrier	*8*	running
running, marathon	*13.3*	running
**Hip-hop dance***	**7.8**	dancing
**Step aerobic***	**7.5**	
aerobic, step, with 6 - 8inch step	*7.5*	dancing
aerobic, step, with 10 - 12inch step	*9.5*	dancing

aerobic, step, with 4-inch step	*5.5*	dancing
**Circuit boxing***	**7.8**	
boxing, in ring, general	*12.8*	sports
boxing, punching bag	*5.5*	sports
boxing, sparring	*7.8*	sports
**Pilates***	**3**	conditioning exercise
**squash***	**9.65**	
squash (Taylor Code 530)	*12*	sports
squash, general	*7.3*	sports
**Reebok Coreboard/ Off-ice skating***	**3.9**	
calisthenics (e.g., situps, abdominal crunches), light effort	*2.8*	conditioning exercise
resistance (weight) training, squats, slow or explosive effort	*5*	conditioning exercise
**Abs**	**2.8**	conditioning exercise
**x-trainer***	**6.8**	
Elliptical trainer, moderate effort	*5*	conditioning exercise
bicycling, stationary, general	*7*	conditioning exercise
bicycling, stationary, 30-50 watts, very light to light effort	*3.5*	conditioning exercise
bicycling, stationary, 90-100 watts, moderate to vigorous effort	*6.8*	conditioning exercise
bicycling, stationary, 101-160 watts, vigorous effort	*8.8*	conditioning exercise
bicycling, stationary, 161-200 watts, vigorous effort	*11*	conditioning exercise
bicycling, stationary, 51-89 watts, light-to-moderate effort	*4.8*	conditioning exercise
**Cardio***	**9**	
stair-treadmill ergometer, general	*9*	conditioning exercise
rope skipping, general	*12.3*	conditioning exercise
bicycling, stationary, general	*7*	conditioning exercise
bicycling, stationary, 30-50 watts, very light to light effort	*3.5*	conditioning exercise
bicycling, stationary, 90-100 watts, moderate to vigorous effort	*6.8*	conditioning exercise
bicycling, stationary, 101-160 watts, vigorous effort	*8.8*	conditioning exercise
bicycling, stationary, 161-200 watts, vigorous effort	*11*	conditioning exercise
bicycling, stationary, 201-270 watts, very vigorous effort	*14*	conditioning exercise
bicycling, stationary, 51-89 watts, light-to-moderate effort	*4.8*	conditioning exercise
bicycling, stationary, RPM/Spin bike class	*8.5*	conditioning exercise
aerobic, general	*7.3*	dancing

aerobic, step, with 6 - 8 inch step	*7.5*	dancing
aerobic, step, with 10 - 12 inch step	*9.5*	dancing
aerobic, step, with 4-inch step	*5.5*	dancing
bench step class, general	*8.5*	dancing
aerobic, low impact	*5*	dancing
aerobic, high impact	*7.3*	dancing
jog/walk combination (jogging component of less than 10 minutes) (Taylor Code 180)	*6*	running
jogging, general	*7*	running
jogging, in place	*8*	running
jogging, on a mini-tramp	*4.5*	running
Running, 4 mph (13 min/mile)	*6*	running
running, 5 mph (12 min/mile)	*8.3*	running
running, 5.2 mph (11.5 min/mile)	*9*	running
running, 6 mph (10 min/mile)	*9.8*	running
running, 6.7 mph (9 min/mile)	*10.5*	running
running, 7 mph (8.5 min/mile)	*11*	running
running, 7.5 mph (8 min/mile)	*11.5*	running
running, 8 mph (7.5 min/mile)	*11.8*	running
running, 8.6 mph (7 min/mile)	*12.3*	running
running, 9 mph (6.5 min/mile)	*12.8*	running
running, 10 mph (6 min/mile)	*14.5*	running
running, 11 mph (5.5 min/mile)	*16*	running
running, 12 mph (5 min/mile)	*19*	running
running, 13 mph (4.6 min/mile)	*19.8*	running
running, 14 mph (4.3 min/mile)	*23*	running
running, cross country	*9*	running
running, (Taylor code 200)	*8*	running
running, stairs, up	*15*	running
**Stretch**	**2.3**	conditioning exercise
**Figure Skating**	**14**	winter activities
**Tone***	**3.3**	
calisthenics (e.g., push-ups, sit ups, pull-ups, lunges), moderate effort	*3.8*	conditioning exercise
calisthenics (e.g., sit-ups, abdominal crunches), light effort	*2.8*	conditioning exercise
**Ballet***	**5.9**	

ballet, modern, or jazz, general, rehearsal or class	*5*	dancing
ballet, modern, or jazz, performance, vigorous effort	*6.8*	dancing
**Skating***	**8**	
skating, ice, 9 mph or less	*5.5*	winter activities
skating, ice, general (Taylor Code 360)	*7*	winter activities
skating, ice, rapidly, more than 9 mph, not competitive	*9*	winter activities
skating, speed, competitive	*13.3*	winter activities
**Swimming Laps***	**7.8**	
swimming laps, freestyle, fast, vigorous effort	*9.8*	water activities
swimming laps, freestyle, front crawl, slow, light or moderate effort	*5.8*	water activities
**Swimming***	**8**	
swimming laps, freestyle, fast, vigorous effort	*9.8*	water activities
swimming laps, freestyle, front crawl, slow, light or moderate effort	*5.8*	water activities
swimming, backstroke, general, training or competition	*9.5*	water activities
swimming, backstroke, recreational	*4.8*	water activities
swimming, breaststroke, general, training or competition	*10.3*	water activities
swimming, breaststroke, recreational	*5.3*	water activities
swimming, butterfly, general	*13.8*	water activities
swimming, crawl, fast speed, ~75 yards/minute, vigorous effort	*10*	water activities
swimming, crawl, medium speed, ~50 yards/minute, vigorous effort	*8.3*	water activities
swimming, lake, ocean, river (Taylor Codes 280, 295)	*6*	water activities
swimming, leisurely, not lap swimming, general	*6*	water activities
swimming, sidestroke, general	*7*	water activities
swimming, synchronized	*8*	water activities
swimming, treading water, fast, vigorous effort	*9.8*	water activities
swimming, treading water, moderate effort, general	*3.5*	water activities
**Basketball Pickups***	**6.25**	
basketball, non-game, general (Taylor Code 480)	*6*	sports
basketball, general	*6.5*	sports
basketball, shooting baskets	*4.5*	sports
basketball, drills, practice	*9.3*	sports

*Mets score was calculated as the median of exercise subtypes (italic font)

Supplemental Table 4: Gas chromatography-mass spectrometry information of fatty acid methyl esters determined in the study.

Fatty acid methyl ester	Corresponding fatty acid (IUPAC name)	Retention time (minutes)	Quantifier ion (m/z)	Qualifier ion (m/z)	PubChem ID
Methyl miristate	tetradecanoic acid	6.9	74.0	87.0	https://pubchem.ncbi.nlm.nih.gov/compound/31284
Methyl palmitate	hexadecanoic acid	8.0	74.0	87.0	https://pubchem.ncbi.nlm.nih.gov/compound/8181
Methyl palmitoleate	(Z)-hexadec-9-enoic acid	8.2	55.0	74.0	https://pubchem.ncbi.nlm.nih.gov/compound/643801
Methyl stearate	octadecanoic acid	9.3	74.0	87.0	https://pubchem.ncbi.nlm.nih.gov/compound/8201
Methyl elaidate	(E)-octadec-9-enoic acid	9.4	55.0	74.0	https://pubchem.ncbi.nlm.nih.gov/compound/5280590
Methyl oleate	(Z)-octadec-9-enoic acid	9.5	55.0	74.0	https://pubchem.ncbi.nlm.nih.gov/compound/5364509
Methyl linoleate	(9Z,12Z)-octadeca-9,12-dienoic acid	9.9	81.0	67.0	https://pubchem.ncbi.nlm.nih.gov/compound/5284421
Methyl linolenate	(9Z,12Z,15Z)-octadeca-9,12,15-trienoic acid	10.4	79.0	81.0	https://pubchem.ncbi.nlm.nih.gov/compound/5319706
Methyl arachidate	icosanoic acid	10.9	74.0	87.0	https://pubchem.ncbi.nlm.nih.gov/compound/14259
Methyl eicosenoate	icos-9-enoic acid	11.1	55.0	74.0	
Methyl eicosadienoate	(5Z,14Z)-icosa-8,11-dienoic acid	11.6	81.0	67.0	https://pubchem.ncbi.nlm.nih.gov/compound/5365566
Methyl dihomo-gamma-linolenate	(8Z,11Z,14Z)-icosa-8,11,14-trienoic acid	12.0	79.0	81.0	https://pubchem.ncbi.nlm.nih.gov/compound/5363092
Methyl arachidonate	(5Z,8Z,11Z,14Z)-icosa-5,8,11,14-tetraenoic acid	12.2	79.0	91.0	https://pubchem.ncbi.nlm.nih.gov/compound/6421258
Methyl behenate	docosanoic acid	12.7	74.0	87.0	https://pubchem.ncbi.nlm.nih.gov/compound/13584
Methyl eicosapentaenoate	(5Z,8Z,11Z,14Z,17Z)-icosa-5,8,11,14,17-pentaenoic acid	12.8	79.0	91.0	https://pubchem.ncbi.nlm.nih.gov/compound/13829678
Methyl lignocerate	Tetracosanoic acid	14.7	74.0	87.0	https://pubchem.ncbi.nlm.nih.gov/compound/75546
Methyl docosahexaenoate	(4Z,7Z,10Z,13Z,16Z,19Z)-docosa-4,7,10,13,16,19-hexaenoic acid	15.1	79.0	91.0	https://pubchem.ncbi.nlm.nih.gov/compound/6421262

References

1. Chan, J.L., Heist, K., DePaoli, A.M., Veldhuis, J.D. & Mantzoros, C.S. The role of falling leptin levels in the neuroendocrine and metabolic adaptation to short-term starvation in healthy men. *J Clin Invest* **111**, 1409-1421 (2003).
2. Chan, J.L.*, et al.* Differential regulation of metabolic, neuroendocrine, and immune function by leptin in humans. *Proc Natl Acad Sci U S A* **103**, 8481-8486 (2006).
3. Chan, J.L.*, et al.* Leptin does not mediate short-term fasting-induced changes in growth hormone pulsatility but increases IGF-I in leptin deficiency states. *J Clin Endocrinol Metab* **93**, 2819-2827 (2008).
4. Pilitsi, E., Peradze, N., Perakakis, N. & Mantzoros, C.S. Circulating levels of the components of the GH/IGF-1/IGFBPs axis total and intact IGF-binding proteins (IGFBP) 3 and IGFBP 4 and total IGFBP 5, as well as PAPPA, PAPPA2 and Stanniocalcin-2 levels are not altered in response to energy deprivation and/or metreleptin administration in humans. *Metabolism* **97**, 32-39 (2019).
5. Kang, E.S., Magkos, F., Sienkiewicz, E. & Mantzoros, C.S. Circulating vaspin and visfatin are not affected by acute or chronic energy deficiency or leptin administration in humans. *Eur J Endocrinol* **164**, 911-917 (2011).
6. Chan, J.L., Wong, S.L. & Mantzoros, C.S. Pharmacokinetics of subcutaneous recombinant methionyl human leptin administration in healthy subjects in the fed and fasting states: regulation by gender and adiposity. *Clin Pharmacokinet* **47**, 753-764 (2008).
7. Chou, S.H.*, et al.* Leptin is an effective treatment for hypothalamic amenorrhea. *Proc Natl Acad Sci U S A* **108**, 6585-6590 (2011).
8. Welt, C.K.*, et al.* Recombinant human leptin in women with hypothalamic amenorrhea. *N Engl J Med* **351**, 987-997 (2004).
9. Chan, J.L., Mietus, J.E., Raciti, P.M., Goldberger, A.L. & Mantzoros, C.S. Short-term fasting-induced autonomic activation and changes in catecholamine levels are not mediated by changes in leptin levels in healthy humans. *Clin Endocrinol (Oxf)* **66**, 49-57 (2007).
10. Brinkoetter, M., Magkos, F., Vamvini, M. & Mantzoros, C.S. Leptin treatment reduces body fat but does not affect lean body mass or the myostatin-follistatin-activin axis in lean hypoleptinemic women. *Am J Physiol Endocrinol Metab* **301**, E99-E104 (2011).

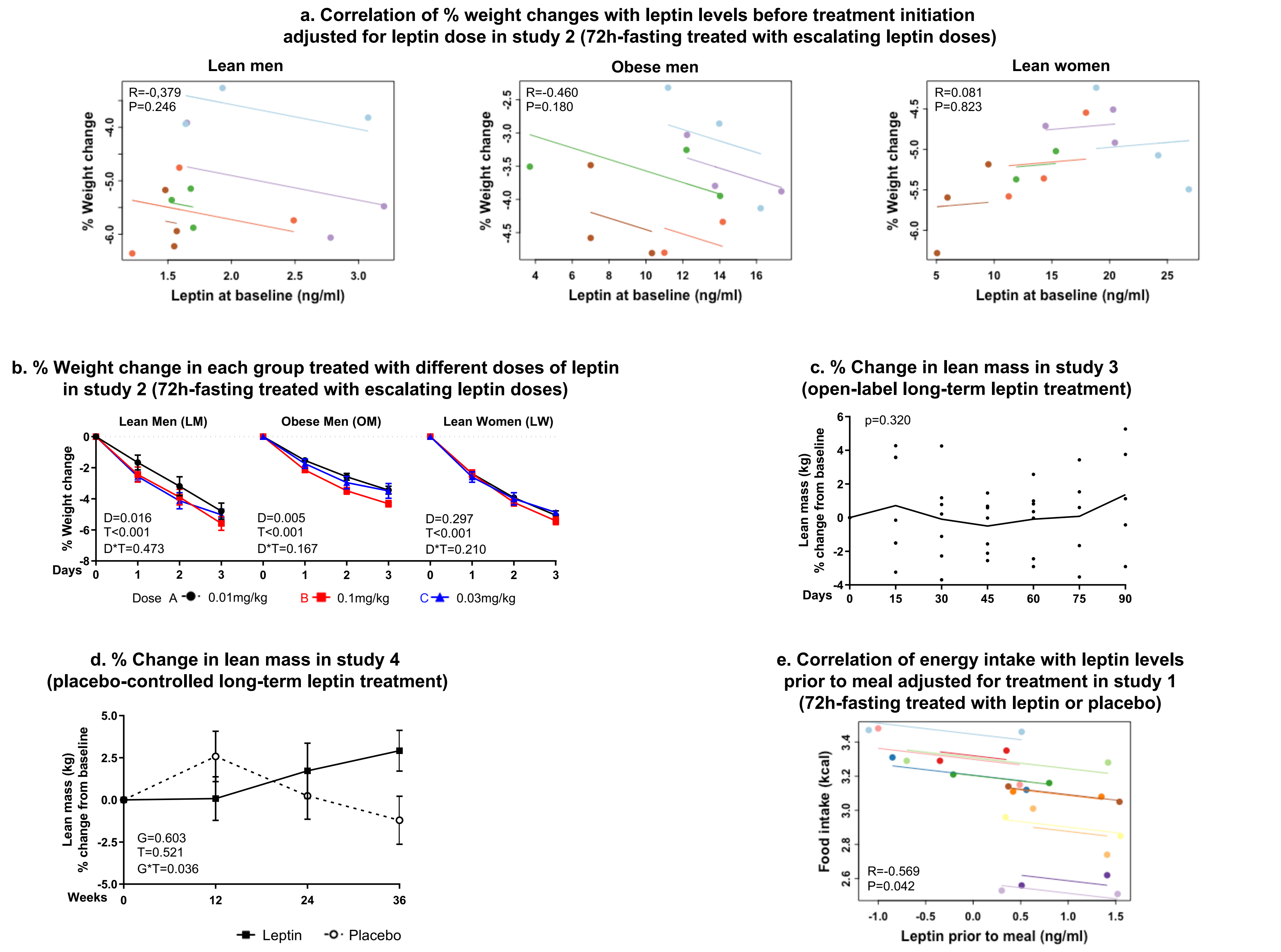
a. Correlation of % weight changes with leptin levels before treatment initiation
adjusted for leptin dose in study 2 (72h-fasting treated with escalating leptin doses)
Lean men
R=-0,379
P=0.246
% Weight change
Leptin at baseline (ng/ml)
Obese men
R=-0.460
P=0.180
% Weight change
Leptin at baseline (ng/ml)
Lean women
R=0.081
P=0.823
% Weight change
Leptin at baseline (ng/ml)
b. % Weight change in each group treated with different doses of leptin
in study 2 (72h-fasting treated with escalating leptin doses)
Lean Men (LM)
Obese Men (OM)
Lean Women (LW)
% Weight change
D=0.016
T<0.001
D*T=0.473
D=0.005
T<0.001
D*T=0.167
D=0.297
T<0.001
D*T=0.210
Days
Dose A 0.01mg/kg
B 0.1mg/kg
C 0.03mg/kg
c. % Change in lean mass in study 3
(open-label long-term leptin treatment)
p=0.320
Lean mass (kg)
% change from baseline
Days
d. % Change in lean mass in study 4
(placebo-controlled long-term leptin treatment)
Lean mass (kg)
% change from baseline
G=0.603
T=0.521
G*T=0.036
Weeks
Leptin
Placebo
e. Correlation of energy intake with leptin levels
prior to meal adjusted for treatment in study 1
(72h-fasting treated with leptin or placebo)
Food intake (kcal)
R=-0.569
P=0.042
Leptin prior to meal (ng/ml)

Supplementary Figure 1

a. Correlation of baseline leptin levels with % weight changes after 72-hour fasting treated with escalating leptin doses (Study 2). Three dots of the same color correspond to the three correlation points one subject contributes to the graph, i.e. one dot for each of the doses. The line refers to the correlation curve that derives from the same colored dots. The reported R is the total, calculated from the individual line curves of each subject and is thus adjusted for the fact that each subject contributed three points (for each leptin dose) in the correlation.

b. Comparison of % weight change between the three leptin doses (Study 2). P-values of D (Dose, i.e. 0.01, 0.1, 0.3 mg/kg/day), T (Time) and D*T interaction of mixed models adjusted for baseline are reported.

c. % changes in lean mass during open-label long-term leptin treatment (Study 3). P-value of time effect of mixed models adjusted for baseline is reported.

d. % changes in lean mass during a placebo-controlled long-term leptin treatment (study 4). Two sided p-values of G (Group, i.e. placebo or leptin), T (Time) and G*T interaction of mixed models are reported. Post-hoc t-tests for the different timepoints by G*T<0.001 were performed but were not significant.

e. Correlation of leptin levels before an ad-libitum meal with energy intake at the meal after 72-hour fasting treated with leptin or placebo (Study 1). Two dots of the same color correspond to the two correlation points that one subject contributes to the graph, i.e. one for placebo and one for leptin treatment. The line refers to the correlation curve that derives from the two same-colored dots. The reported R is the total, calculated from the individual line curves of each subject and is thus adjusted for the fact that each subject contributed two points (placebo and leptin) in the correlation. Energy intake and leptin values were logarithmically transformed to improve linearity for performing the repeated measures analysis. In a and e R and p-value were calculated with rmcorr package in R studio. In b and d data are presented as Means ± SEMs.

Exact p-values

Sup. Figure 1b: Lean Men T=0.005×10^{-21}; Obese Men T=0.001×10^{-15}; Lean Women T=0.008×10^{-18}

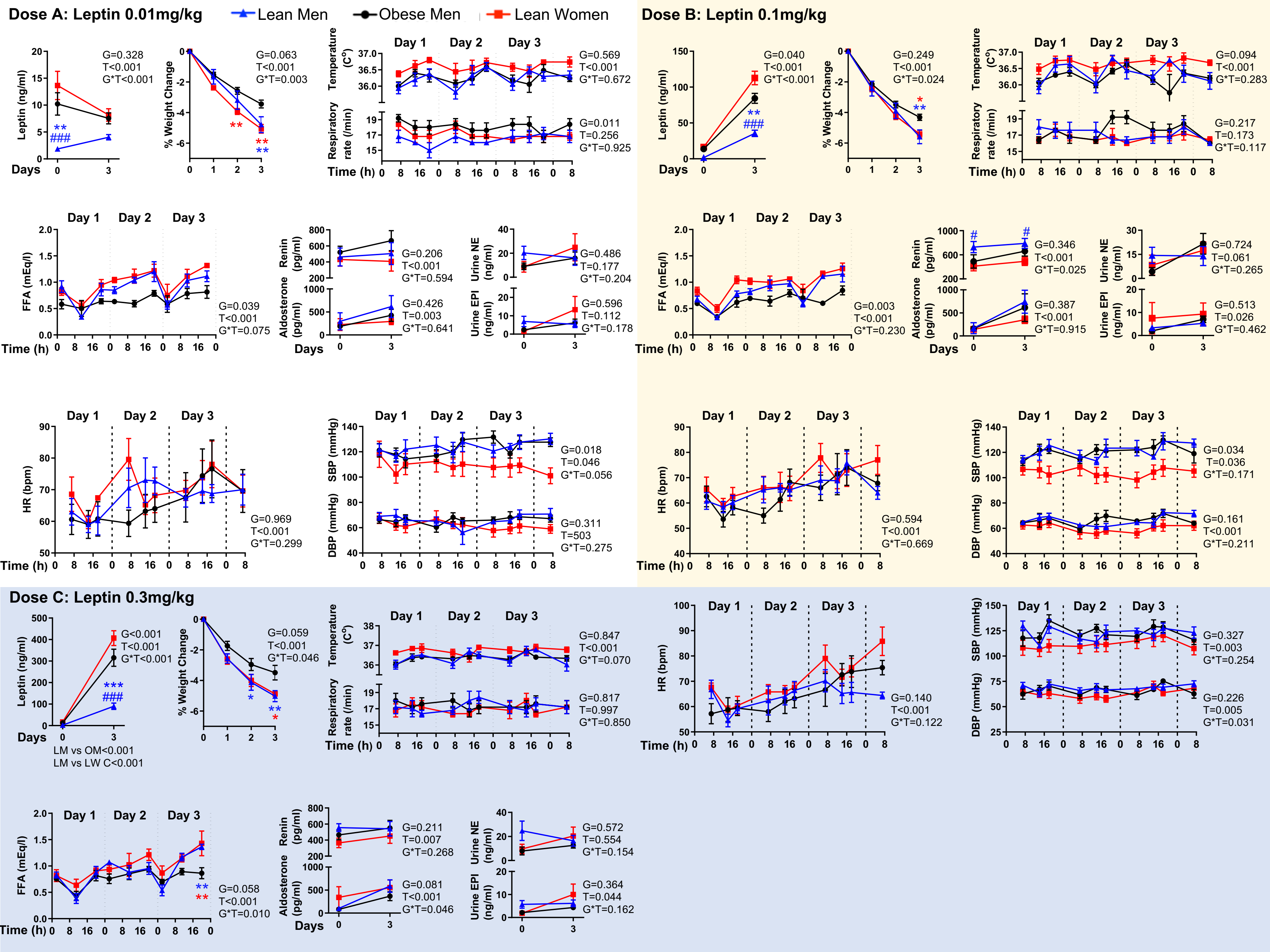
Dose A: Leptin 0.01mg/kg
Lean Men
Obese Men
Lean Women
Leptin (ng/ml)
Days
G=0.328
T<0.001
G*T<0.001
**
###
% Weight Change
G=0.063
T<0.001
G*T=0.003
**
**
**
Temperature (C°)
Respiratory rate (/min)
Day 1
Day 2
Day 3
Time (h)
G=0.569
T<0.001
G*T=0.672
G=0.011
T=0.256
G*T=0.925
FFA (mEq/l)
G=0.039
T<0.001
G*T=0.075
Renin (pg/ml)
G=0.206
T<0.001
G*T=0.594
Aldosterone (pg/ml)
G=0.426
T=0.003
G*T=0.641
Urine NE (ng/ml)
G=0.486
T=0.177
G*T=0.204
Urine EPI (ng/ml)
G=0.596
T=0.112
G*T=0.178
HR (bpm)
G=0.969
T<0.001
G*T=0.299
SBP (mmHg)
G=0.018
T=0.046
G*T=0.056
DBP (mmHg)
G=0.311
T=503
G*T=0.275
Dose B: Leptin 0.1mg/kg
Leptin (ng/ml)
G=0.040
T<0.001
G*T<0.001
**
###
% Weight Change
G=0.249
T<0.001
G*T=0.024
*
**
Temperature (C°)
G=0.094
T<0.001
G*T=0.283
Respiratory rate (/min)
G=0.217
T=0.173
G*T=0.117
FFA (mEq/l)
G=0.003
T<0.001
G*T=0.230
Renin (pg/ml)
#
#
G=0.346
T<0.001
G*T=0.025
Aldosterone (pg/ml)
G=0.387
T<0.001
G*T=0.915
Urine NE (ng/ml)
G=0.724
T=0.061
G*T=0.265
Urine EPI (ng/ml)
G=0.513
T=0.026
G*T=0.462
HR (bpm)
G=0.594
T<0.001
G*T=0.669
SBP (mmHg)
G=0.034
T=0.036
G*T=0.171
DBP (mmHg)
G=0.161
T<0.001
G*T=0.211
Dose C: Leptin 0.3mg/kg
Leptin (ng/ml)
G<0.001
T<0.001
G*T<0.001
***
###
LM vs OM<0.001
LM vs LW C<0.001
% Weight Change
G=0.059
T<0.001
G*T=0.046
*
**
*
Temperature (C°)
G=0.847
T<0.001
G*T=0.070
Respiratory rate (/min)
G=0.817
T=0.997
G*T=0.850
HR (bpm)
G=0.140
T<0.001
G*T=0.122
SBP (mmHg)
G=0.327
T=0.003
G*T=0.254
DBP (mmHg)
G=0.226
T=0.005
G*T=0.031
FFA (mEq/l)
**
**
G=0.058
T<0.001
G*T=0.010
Renin (pg/ml)
G=0.211
T=0.007
G*T=0.268
Aldosterone (pg/ml)
G=0.081
T<0.001
G*T=0.046
Urine NE (ng/ml)
G=0.572
T=0.554
G*T=0.154
Urine EPI (ng/ml)
G=0.364
T=0.044
G*T=0.162

Supplementary Figure 2: Comparison of the effects of escalating doses of leptin during 72-hour fasting between lean men, obese men and lean women (Study 2; n=15 [5 lean men, 5 obese men and 5 lean women]).
White panel corresponds to physiologic dose A, beige panel to supraphysiologic dose B and blue panel to pharmacologic dose C. Leptin levels, % weight change, respiratory rate, body temperature, free fatty acids (FFA), aldosterone, renin, urine epinephrine (EPI), urine norepinephrine (NE), heart rate (HR), systolic (SBP), and diastolic (DBP) blood pressure, are demonstrated in each panel. Means ± SEMs are demonstrated. Two-sides p-values of G (Group, i.e. lean men vs obese men vs lean women), T (Time, i.e. days/hours of fasting) and G*T (Group with Time interaction) of mixed model analysis adjusted for baseline are reported. By p of G or G*T<0.05, post-hoc Bonferroni test was performed both between the estimated means of the three groups and between the three groups at each timepoint. One, two or three asterisks correspond to p<0.05, <0.01 or <0.001, respectively. Asterisks are blue for lean men vs obese men and red for lean women vs obese men; Three blue hash signs correspond to p<0.001 for lean men vs lean women. LM: Lean Men; OM: Obese Men; LW: Lean Women.

Exact p-values

Dose A : Leptin $T=0.001\times10^{-8}$; $G*T=0.007\times10^{-2}$; Baseline LMvsOM=0.006 & LMvsLW=0.001×10^{-1}. **Weight Change%** $T=0.001\times10^{-21}$; Day 2 LWvsOM=0.009; Day 3 LMvsOM=0.009; Day 3 LWvsOM=0.002. **Temperature** $T=0.003\times10^{-2}$. **FFA** $T=0.005\times10^{-10}$. **Renin** $T=0.004\times10^{-3}$. **HR** $T=0.002\times10^{-1}$

Dose B: Leptin $T=0.004\times10^{-13}$; $G*T=0.002\times10^{-2}$; Day 3 LMvsOM=0.005; Day 3 LMvsLW=0.001×10^{-1}. **Weight Change%** $T=0.006\times10^{-25}$; Day 3 LWvsOM=0.026; Day 3 LMvsOM=0.001. **Temperature** $T=0.003\times10^{-2}$. **FFA** $T=0.002\times10^{-11}$. **Aldosterone** $T=0.002\times10^{-3}$. **Renin** $T=0.001\times10^{-9}$; Baseline LMvsLW=0.015; Day 3 LMvsLW=0.025. **HR** $T=0.004\times10^{-4}$. **DBP** $T=0.003\times10^{-1}$

Dose C: Leptin $G=0.001\times10^{-1}$; $T=0.002\times10^{-16}$; $G*T=0.001\times10^{-5}$; LMvsOM=0.001×10^{-1}; LMvsLW=0.001×10^{-1}; Day 3 LMvsOM=0.005×10^{-1}; Day 3 LMvsLW=0.001×10^{-1}**. Weight Change%** $T=0.009\times10^{-10}$; Day 2 LMvsOM=0.037; Day 3 LMvsOM=0.009; Day 3 LWvsOM=0.018. **Temperature** $T=0.002\times10^{-1}$. **FFA** $T=0.001\times10^{-10}$; Day 3 (16hrs) LMvsOM=0.002 & LWvsOM=0.002. **Aldosterone** $T=0.003\times10^{-7}$. **HR** $T=0.007\times10^{-6}$

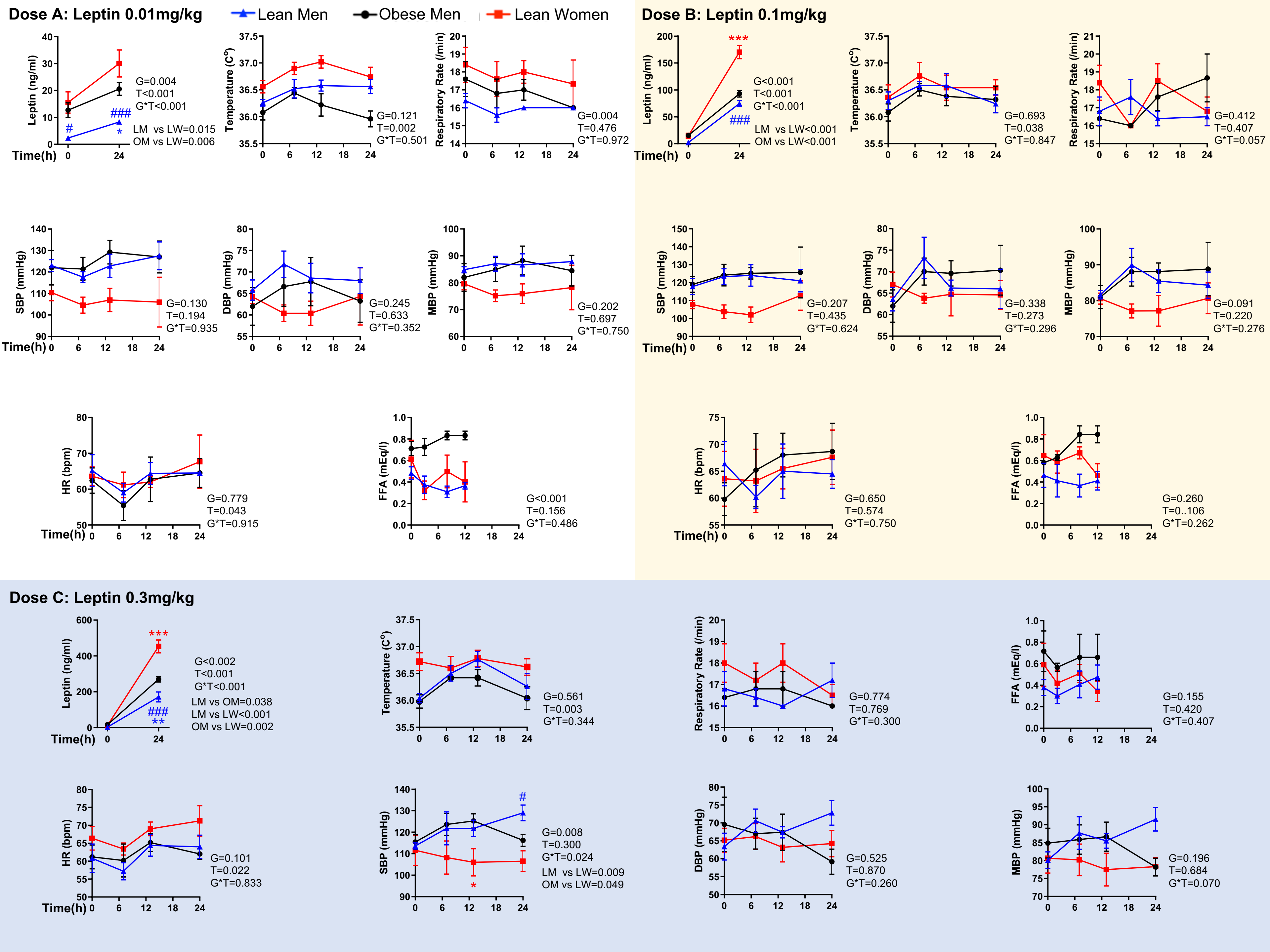

Dose A: Leptin 0.01mg/kg
Lean Men
Obese Men
Lean Women
Dose B: Leptin 0.1mg/kg
Dose C: Leptin 0.3mg/kg
Leptin (ng/ml)
Temperature (C°)
Respiratory Rate (/min)
SBP (mmHg)
DBP (mmHg)
MBP (mmHg)
HR (bpm)
FFA (mEq/l)
Time(h)
G=0.004 T<0.001 G*T<0.001
LM vs LW=0.015
OM vs LW=0.006
G=0.121 T=0.002 G*T=0.501
G=0.004 T=0.476 G*T=0.972
G<0.001 T<0.001 G*T<0.001
LM vs LW<0.001
OM vs LW<0.001
G=0.693 T=0.038 G*T=0.847
G=0.412 T=0.407 G*T=0.057
G=0.130 T=0.194 G*T=0.935
G=0.245 T=0.633 G*T=0.352
G=0.202 T=0.697 G*T=0.750
G=0.207 T=0.435 G*T=0.624
G=0.338 T=0.273 G*T=0.296
G=0.091 T=0.220 G*T=0.276
G=0.779 T=0.043 G*T=0.915
G<0.001 T=0.156 G*T=0.486
G=0.650 T=0.574 G*T=0.750
G=0.260 T=0..106 G*T=0.262
G<0.002 T<0.001 G*T<0.001
LM vs OM=0.038
LM vs LW<0.001
OM vs LW=0.002
G=0.561 T=0.003 G*T=0.344
G=0.774 T=0.769 G*T=0.300
G=0.155 T=0.420 G*T=0.407
G=0.101 T=0.022 G*T=0.833
G=0.008 T=0.300 G*T=0.024
LM vs LW=0.009
OM vs LW=0.049
G=0.525 T=0.870 G*T=0.260
G=0.196 T=0.684 G*T=0.070

Supplementary Figure 3: Comparison of the effects of a single administration of escalating doses of leptin in the 24-hour fed state between lean men, obese men and lean women (Study 2; n=15 [5 lean men, 5 obese men and 5 lean women]).
White panel corresponds to physiologic dose A, beige panel to supraphysiologic dose B and blue panel to pharmacologic dose C. Leptin levels, body temperature, respiratory rate, systolic (SBP), diastolic (DBP), mean (MBP) blood pressure, heart rate (HR), and free fatty acids (FFA) are demonstrated in each panel. Means ± SEMs are demonstrated. Mixed model analysis was performed adjusted for baseline. Two-sided p-values of G (Group, i.e. lean men vs obese men vs lean women), T (Time, i.e. hours from leptin administration) and G*T (Group with Time interaction) of mixed model analysis adjusted for baseline are reported. By p of G or G*T<0.05, post-hoc Bonferroni test was performed both between the estimated means of the three groups and between the three groups at each timepoint. One, two or three asterisks correspond to p<0.05, <0.01 or <0.001, respectively. Asterisks are blue for lean men vs obese men and red for lean women vs obese men; One or three blue hash signs correspond to p<0.05 or <0.001, respectively for lean men vs lean women. LM: Lean Men; OM: Obese Men; LW: Lean Women.

Exact p-values
Dose A: Leptin T=0.003×10^{-6}; G*T=0.003×10^{-1}; Baseline LMvsLW=0.015; Day1 (24hrs) LMvsOM=0.025 & LMvsLW=0.001×10^{-1}. **FFA** G=0.004×10^{-30}
Dose B: Leptin G=0.003×10^{-2}; T=0.008×10^{-8}; G*T=0.002×10^{-2}; LMvsLW=0.001×10^{-1}; LWvsOM=0.001×10^{-1}; Day 1 (24hrs) LMvsLW=0.001×10^{-1} & LWvsOM=0.001×10^{-1}
Dose C: Leptin G=0.002×10^{-2}; T=0.001×10^{-6}; G*T=0.007×10^{-2}; LMvsLW=0.001×10^{-1}; Day 1 (24hrs) LMvsOM=0.005 & LWvsOM=0.001×10^{-1} & LMvsLW=0.001×10^{-1}

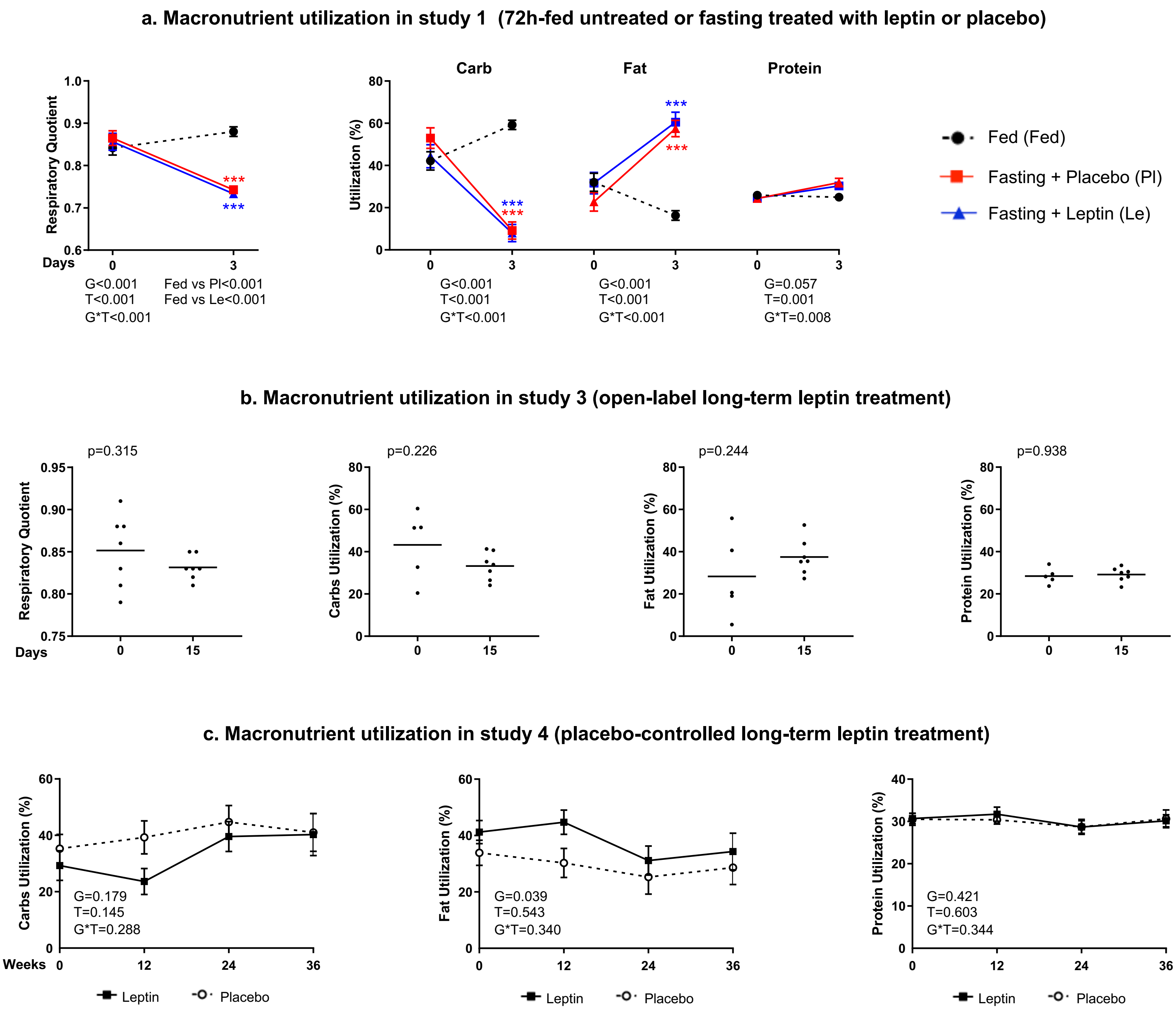
a. Macronutrient utilization in study 1 (72h-fed untreated or fasting treated with leptin or placebo)
Respiratory Quotient
Days
G<0.001
T<0.001
G*T<0.001
Fed vs Pl<0.001
Fed vs Le<0.001
Carb
Fat
Protein
Utilization (%)
G<0.001
T<0.001
G*T<0.001
G<0.001
T<0.001
G*T<0.001
G=0.057
T=0.001
G*T=0.008
Fed (Fed)
Fasting + Placebo (Pl)
Fasting + Leptin (Le)
b. Macronutrient utilization in study 3 (open-label long-term leptin treatment)
p=0.315
p=0.226
p=0.244
p=0.938
Respiratory Quotient
Carbs Utilization (%)
Fat Utilization (%)
Protein Utilization (%)
Days
c. Macronutrient utilization in study 4 (placebo-controlled long-term leptin treatment)
Carbs Utilization (%)
G=0.179
T=0.145
G*T=0.288
Fat Utilization (%)
G=0.039
T=0.543
G*T=0.340
Protein Utilization (%)
G=0.421
T=0.603
G*T=0.344
Weeks
Leptin
Placebo

Supplementary Figure 4: **Short and long-term leptin effects on respiratory quotient and macronutrient utilization.**
a. 72-hour isocaloric fed state (untreated) or fasting treated with leptin or placebo (Study 1, n=13). *Left:* Respiratory quotient and *Right:* macronutrient utilization based on respiratory quotient at start and treatment completion. Means ± SEMs are demonstrated. Mixed model was performed adjusted for baseline. Two-sided p-values of G (Group, i.e. fed untreated or fasting treated with placebo or leptin), T (Time, i.e. days of study) and G*T (interaction of Group with Time) of mixed models are reported. By p of G*T<0.05, post-hoc Bonferroni test was performed between the estimated means of the three groups (only significant results are demonstrated) and between the three groups at each timepoint. Three asterisks indicate p<0.001 in the Bonferroni post-hoc test. Asterisks are red for fed vs fasting+placebo and blue for fed vs fasting+leptin. b. Open-label long-term leptin treatment in mildly hypoleptinemic women (Study 3, n=7). Respiratory quotient and calculation of utilization of carbohydrates, fats and proteins based on respiratory quotient (from left to right). Two-sided p-value of paired t-test is reported for respiratory quotient and macronutrient utilization. c. Placebo-controlled long-term leptin treatment in mildly hypoleptinemic women (Study 4, n=19 [leptin group=10; placebo group=9]). Calculation of utilization of carbohydrates, fats and proteins (from left to right) based on respiratory quotient (previously published in [30]). P-values of G (Group, i.e. placebo or leptin), T (Time, i.e. weeks of study) and G*T (interaction of Group with Time) of mixed models are reported.

Exact p-values
RQ G=0.001×10^{-3}; T=0.003×10^{-9}; G*T=0.007×10^{-10}; Fed vs Pl=0.002×10^{-2}; Fed vs Le=0.003×10^{-7}; Day 3 Fed vs Pl=0.001×10^{-12} & Fed vs Le=0.003×10^{-13}. **Carb Utilization (%)** G=0.004×10^{-11}; T=0.004×10^{-9}; G*T=0.003×10^{-12}; Fed vs Pl=0.007×10^{-10}; Fed vs Le=0.001×10^{-8}; Day 3 Fed vs Pl=0.004×10^{-12} & Fed vs Le=0.003×10^{-13}. **Fat Utilization (%)** G=0.003×10^{-7}; T=0.001×10^{-4}; G*T=0.002×10^{-8}; Fed vs Pl=0.004×10^{-6}; Fed vs Le=0.002×10^{-5}; Day 3 Fed vs Pl=0.002×10^{-11} & Fed vs Le=0.005×10^{-9}.

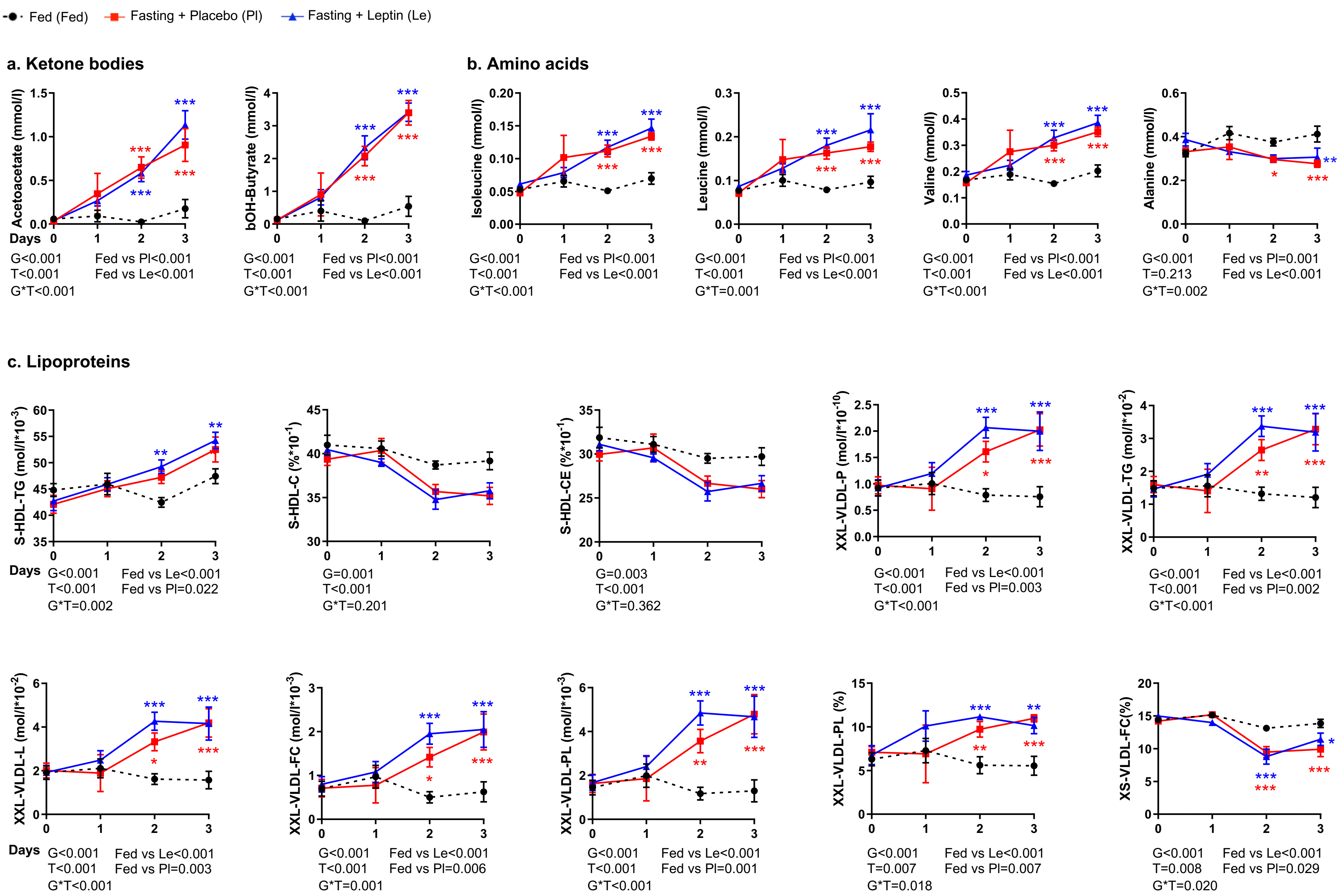
Fed (Fed)
Fasting + Placebo (Pl)
Fasting + Leptin (Le)
a. Ketone bodies
Acetoacetate (mmol/l)
Days
G<0.001
T<0.001
G*T<0.001
Fed vs Pl<0.001
Fed vs Le<0.001
bOH-Butyrate (mmol/l)
G<0.001
T<0.001
G*T<0.001
Fed vs Pl<0.001
Fed vs Le<0.001
b. Amino acids
Isoleucine (mmol/l)
G<0.001
T<0.001
G*T<0.001
Fed vs Pl<0.001
Fed vs Le<0.001
Leucine (mmol/l)
G<0.001
T<0.001
G*T=0.001
Fed vs Pl<0.001
Fed vs Le<0.001
Valine (mmol/l)
G<0.001
T<0.001
G*T<0.001
Fed vs Pl<0.001
Fed vs Le<0.001
Alanine (mmol/l)
G<0.001
T=0.213
G*T=0.002
Fed vs Pl=0.001
Fed vs Le<0.001
c. Lipoproteins
S-HDL-TG (mol/l*10^-3)
Days
G<0.001
T<0.001
G*T=0.002
Fed vs Le<0.001
Fed vs Pl=0.022
S-HDL-C (%*10^-1)
G=0.001
T<0.001
G*T=0.201
S-HDL-CE (%*10^-1)
G=0.003
T<0.001
G*T=0.362
XXL-VLDL-P (mol/l*10^-10)
G<0.001
T<0.001
G*T<0.001
Fed vs Le<0.001
Fed vs Pl=0.003
XXL-VLDL-TG (mol/l*10^-2)
G<0.001
T<0.001
G*T<0.001
Fed vs Le<0.001
Fed vs Pl=0.002
XXL-VLDL-L (mol/l*10^-2)
Days
G<0.001
T<0.001
G*T<0.001
Fed vs Le<0.001
Fed vs Pl=0.003
XXL-VLDL-FC (mol/l*10^-3)
G<0.001
T<0.001
G*T=0.001
Fed vs Le<0.001
Fed vs Pl=0.006
XXL-VLDL-PL (mol/l*10^-3)
G<0.001
T<0.001
G*T<0.001
Fed vs Le<0.001
Fed vs Pl=0.001
XXL-VLDL-PL (%)
G<0.001
T=0.007
G*T=0.018
Fed vs Le<0.001
Fed vs Pl=0.007
XS-VLDL-FC(%)
G<0.001
T=0.008
G*T=0.020
Fed vs Le<0.001
Fed vs Pl=0.029

Supplementary Figure 5: Effects on ketone bodies, amino acids and lipoprotein profile of 72-hour isocaloric fed state (untreated) or fasting treated with leptin or placebo (Study 1, n=13).

Blood concentrations of a. ketone bodies, b. amino acids and c. circulating profile of the ten most important lipoproteins according to sPLS-DA and one-way ANOVA; Means ± SEMs are demonstrated. NMR - based metabolomics were used for quantification in serum samples. Mixed model was performed adjusted for baseline. P-values of G (Group, i.e. fed untreated or fasting treated with placebo or leptin), T (Time, i.e. days of study) and G*T (interaction of Group with Time) of mixed models are reported. By p of G*T<0.05, post-hoc Bonferroni test was performed between the estimated means of the three groups (only significant results are demonstrated) and between the three groups at each timepoint. One, two or three asterisks indicate p<0.05, <0.01 or <0.001, respectively in the Bonferroni post-hoc t-test. Asterisks are red for fed vs fasting+placebo and blue for fed vs fasting+leptin. VLDL, very low density lipoprotein; LDL, low density lipoprotein; IDL, intermediate density lipoprotein; HDL, high density lipoprotein; XS, S, M, L, XL, XXL indicates consecutive size of lipoprotein particles, i.e. very small, small, medium, large, very large, or very very large; P, Concentration of Particles; C, cholesterol; CE, cholesterol esters; FC, free cholesterol; PL, phospholipids; TG, triglycerides; (%) indicates % of the parameter in relation to total lipids.

Exact p-values

Acetoacetate G=0.006×10^{-6}; T=0.004×10^{-11}; G*T=0.001×10^{-2}; Fed vs Pl= 0.006×10^{-3}; Fed vs Le=0.003×10^{-5}; Day 2 Fed vs Pl=0.001×10^{-1} & Fed vs Le=0.001×10^{-1}; Day 3 Fed vs Pl=0.001×10^{-1} & Fed vs Le=0.001×10^{-1}

bOH-Butyrate G=0.003×10^{-12}; T=0.002×10^{-20}; G*T=0.004×10^{-8}; Fed vs Pl= 0.003×10^{-11}; Fed vs Le=0.008×10^{-8}; Day 2 Fed vs Pl=0.001×10^{-1} & Fed vs Le=0.001×10^{-1}; Day 3 Fed vs Pl=0.001×10^{-1} & Fed vs Le=0.001×10^{-1}

Isoleucine G=0.001×10^{-10}; T=0.005×10^{-12}; G*T=0.002×10^{-4}; Fed vs Pl= 0.003×10^{-4}; Fed vs Le=0.003×10^{-5}; Day 2 Fed vs Pl=0.001×10^{-1} & Fed vs Le=0.001×10^{-1}; Day 3 Fed vs Pl=0.001×10^{-1} & Fed vs Le=0.001×10^{-1}

Leucine G=0.005×10^{-7}; T=0.001×10^{-6}; G*T=0.007×10^{-2}; Fed vs Pl= 0.001×10^{-1}; Fed vs Le=0.005×10^{-5}; Day 2 Fed vs Pl=0.002×10^{-1} & Fed vs Le=0.001×10^{-1}; Day 3 Fed vs Pl=0.004×10^{-1} & Fed vs Le=0.001×10^{-1}

Valine G=0.002×10^{-10}; T=0.008×10^{-10}; G*T=0.006×10^{-5}; Fed vs Pl= 0.002×10^{-4}; Fed vs Le=0.007×10^{-8}; Day 2 Fed vs Pl=0.001×10^{-1} & Fed vs Le=0.001×10^{-1}; Day 3 Fed vs Pl=0.001×10^{-1} & Fed vs Le=0.001×10^{-1}

Alanine G=0.001×10^{-2}; Fed vs Le=0.009×10^{-2}; Day 2 Fed vs Pl=0.046×10^{-1}; Day 3 Fed vs Pl=0.002×10^{-1} & Fed vs Le=0.006

S-HDL-TG G=0.005×10^{-1}; T=0.005×10^{-8}; Fed vs Pl= 0.022; Fed vs Le=0.005×10^{-1}; Day 2 Fed vs Le=0.004; Day 3 Fed vs Le=0.005

S-HDL-C(%) T=0.006×10^{-7}

S-HDL-CE(%) T=0.007×10^{-6}

XXL-VLDL-P G=0.003×10^{-4}; T=0.001×10^{-2}; G*T=0.001×10^{-1}; Fed vs Pl= 0.003; Fed vs Le=0.003×10^{-4}; Day 2 Fed vs Pl=0.017 & Fed vs Le=0.001×10^{-1}; Day 3 Fed vs Pl=0.001×10^{-1} & Fed vs Le=0.001×10^{-1}

XXL-VLDL-TG G=0.002×10^{-4}; T=0.005×10^{-3}; G*T=0.001×10^{-1}; ; Fed vs Pl= 0.002; Fed vs Le=0.002×10^{-4}; Day 2 Fed vs Pl=0.005 & Fed vs Le=0.001×10^{-1}; Day 3 Fed vs Pl=0.001×10^{-1} & Fed vs Le=0.001×10^{-1}

XXL-VLDL-L G=0.005×10^{-4}; T=0.002×10^{-2}; G*T=0.002×10^{-1}; Fed vs Le=0.004×10^{-4}; Day 2 Fed vs Pl=0.019 & Fed vs Le=0.001×10^{-1}; Day 3 Fed vs Pl=0.002×10^{-1} & Fed vs Le=0.001×10^{-1}

XXL-VLDL-FC G=0.006×10^{-3}; T=0.003×10^{-2} ; Fed vs Le=0.005×10^{-3}; Day 2 Fed vs Pl=0.029 & Fed vs Le=0.001×10^{-1}; Day 3 Fed vs Pl=0.002×10^{-1} & Fed vs Le=0.006×10^{-1}

XXL-VLDL-PL G=0.005×10^{-4}; T=0.002×10^{-3}; G*T=0.002×10^{-1}; Fed vs Le=0.005×10^{-3}; Day 2 Fed vs Pl=0.007 & Fed vs Le=0.001×10^{-1}; Day 3 Fed vs Pl=0.001×10^{-1} & Fed vs Le=0.001×10^{-1}

XXL-VLDL-PL(%) G=0.003×10^{-3}; Fed vs Le=0.002×10^{-3}; Day 2 Fed vs Pl=0.001 & Fed vs Le=0.002×10^{-1}; Day 3 Fed vs Pl=0.005×10^{-1} & Fed vs Le=0.003

XXL-VLDL-FC(%) G=0.003×10^{-1}; Fed vs Le=0.003×10^{-1}; Day 2 Fed vs Pl=0.008×10^{-1} & Fed vs Le=0.001×10^{-1}; Day 3 Fed vs Pl=0.004×10^{-1} & Fed vs Le=0.041

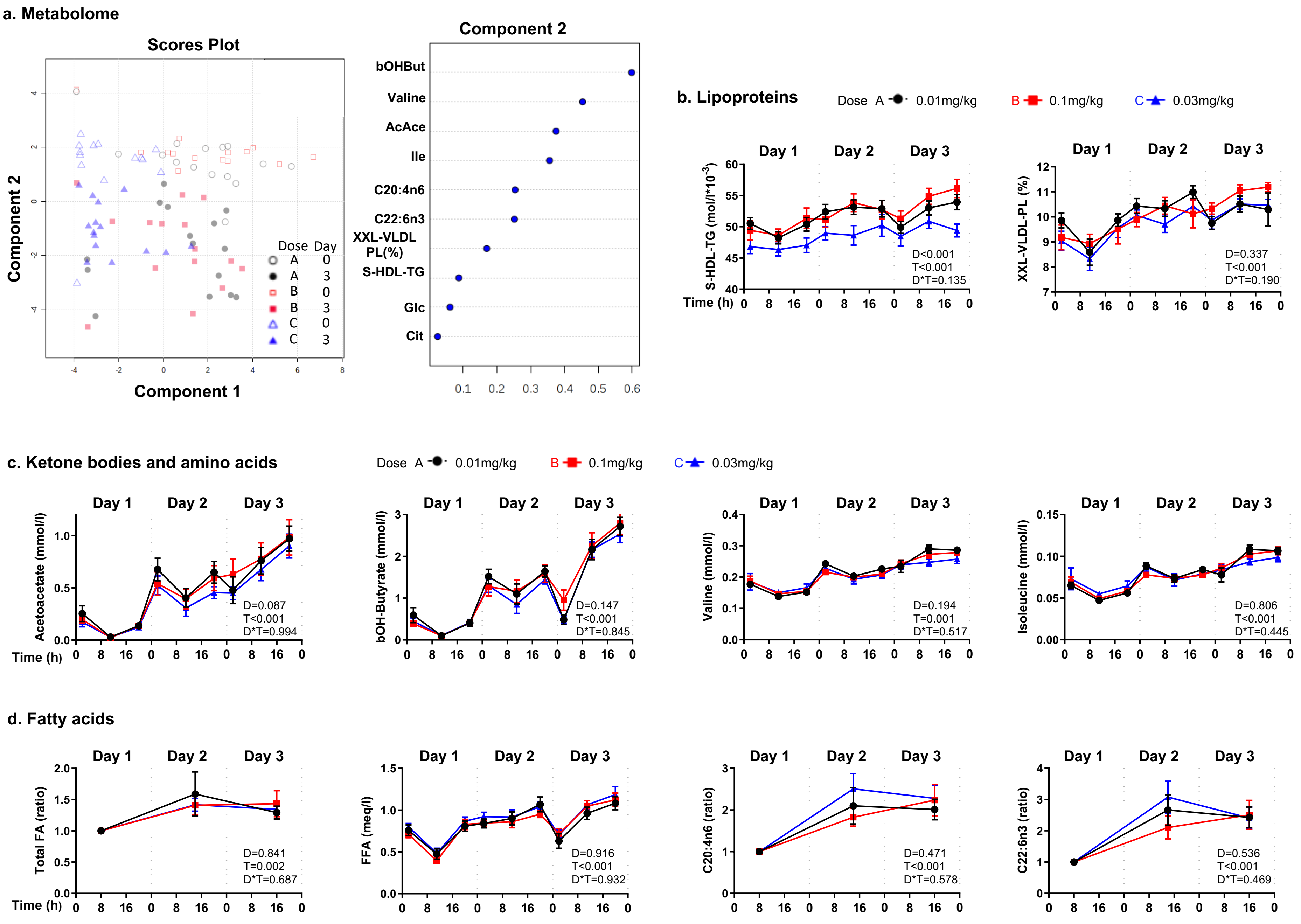

a. Metabolome
Scores Plot
Component 1
Component 2
Dose Day
A 0
A 3
B 0
B 3
C 0
C 3
Component 2
bOHBut
Valine
AcAce
Ile
C20:4n6
C22:6n3
XXL-VLDL PL(%)
S-HDL-TG
Glc
Cit
b. Lipoproteins
Dose A 0.01mg/kg B 0.1mg/kg C 0.03mg/kg
Day 1 Day 2 Day 3
S-HDL-TG (mol/l*10^-3)
D<0.001 T<0.001 D*T=0.135
XXL-VLDL-PL (%)
D=0.337 T<0.001 D*T=0.190
Time (h)
c. Ketone bodies and amino acids
Dose A 0.01mg/kg B 0.1mg/kg C 0.03mg/kg
Acetoacetate (mmol/l)
D=0.087 T<0.001 D*T=0.994
bOH-Butyrate (mmol/l)
D=0.147 T<0.001 D*T=0.845
Valine (mmol/l)
D=0.194 T=0.001 D*T=0.517
Isoleucine (mmol/l)
D=0.806 T<0.001 D*T=0.445
d. Fatty acids
Total FA (ratio)
D=0.841 T=0.002 D*T=0.687
FFA (meq/l)
D=0.916 T<0.001 D*T=0.932
C20:4n6 (ratio)
D=0.471 T<0.001 D*T=0.578
C22:6n3 (ratio)
D=0.536 T<0.001 D*T=0.469

Supplementary figure 6: Effects on metabolite and lipid metabolism of escalating leptin doses during 72-hour fasting (Study 2, n=15). a. sPLS-DA analysis. Left: Score plot of component 1 and component 2. Symbols indicate the measurement of component 1 in relation to the measurement of component 2 for one subject/on one leptin dose/on one day: Black circles correspond to dose A, red squares to dose B and blue triangles to dose C. Unfilled symbols start of treatment (Day 0) and filled symbols end of treatment (Day 3). Unfilled symbols (start of the treatment) are gathered at the upper half of the plot and filled symbols (end of the treatment) are gathered at the lower half of the plot, showing that component 2 is able to differentiate start from end of treatment (independent of dose) Right: Parameters that compose component 2 and their level of contribution (loading) in the component.

Circulating profile of the ten most important parameters according to sPLS-DA and one-way ANOVA belonging to following categories b. lipoproteins, c. ketone bodies and aminoacids, d. fatty acids. Data are presented as Means ± SEMs.
NMR - based metabolomics were used to quantify amino acids, metabolites, and lipids bound to lipoproteins in serum samples.GC/MS-EI was used to quantify fatty acid methyl esters in whole plasma. P-values of D (Dose, i.e. 0.01, 0.1, 0.3 mg/kg/day), T (Time, i.e. hours of study) and D*T (interaction of Dose with Time) of mixed models adjusted for baseline (except for fatty acids expressed as ratios of baseline) are reported. For sPLS-DA, baseline was considered the earliest available measurements at Day 1 (i.e. 2:00 for lipoproteins and FFA and 8:00 for fatty acids) and as day 3 measurement the 12:00 for lipoproteins and FFA and the 14:00 for fatty acids.

Exact p=values
Sup. Figure 5b: S-HDL-TG D=0.009×10^{-7} & T=0.002×10^{-19}; **XXL-VLDL-PL (%)** T=0.003×10^{-20}
Sup. Figure 5c: Acetoacetate T= 0.002×10^{-49}; **bOH-Butyrate** T=0.006×10^{-80}; **Valine** T=0.005×10^{-51}; **Isoleucine** T= 0.005×10^{-41}
Sup. Figure 5d: FFA T=0.001×10^{-6} ; **C20:4n6(ratio)** T= 0.002×10^{-6} ; **C22:6n3(ratio)** T=0.001×10^{-6}

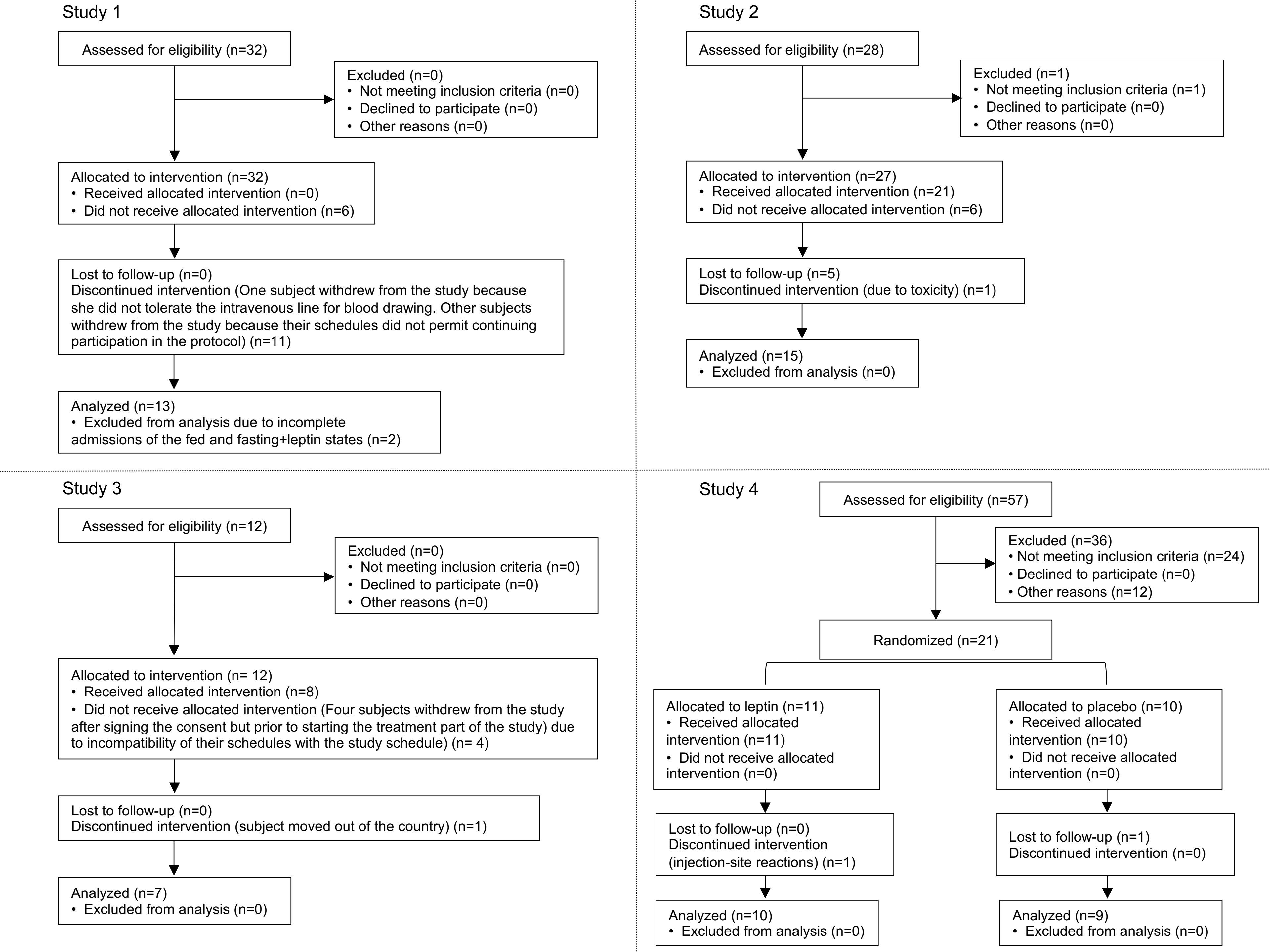

Study 1
Assessed for eligibility (n=32)
Excluded (n=0)
• Not meeting inclusion criteria (n=0)
• Declined to participate (n=0)
• Other reasons (n=0)
Allocated to intervention (n=32)
• Received allocated intervention (n=0)
• Did not receive allocated intervention (n=6)
Lost to follow-up (n=0)
Discontinued intervention (One subject withdrew from the study because she did not tolerate the intravenous line for blood drawing. Other subjects withdrew from the study because their schedules did not permit continuing participation in the protocol) (n=11)
Analyzed (n=13)
• Excluded from analysis due to incomplete admissions of the fed and fasting+leptin states (n=2)
Study 2
Assessed for eligibility (n=28)
Excluded (n=1)
• Not meeting inclusion criteria (n=1)
• Declined to participate (n=0)
• Other reasons (n=0)
Allocated to intervention (n=27)
• Received allocated intervention (n=21)
• Did not receive allocated intervention (n=6)
Lost to follow-up (n=5)
Discontinued intervention (due to toxicity) (n=1)
Analyzed (n=15)
• Excluded from analysis (n=0)
Study 3
Assessed for eligibility (n=12)
Excluded (n=0)
• Not meeting inclusion criteria (n=0)
• Declined to participate (n=0)
• Other reasons (n=0)
Allocated to intervention (n= 12)
• Received allocated intervention (n=8)
• Did not receive allocated intervention (Four subjects withdrew from the study after signing the consent but prior to starting the treatment part of the study) due to incompatibility of their schedules with the study schedule) (n= 4)
Lost to follow-up (n=0)
Discontinued intervention (subject moved out of the country) (n=1)
Analyzed (n=7)
• Excluded from analysis (n=0)
Study 4
Assessed for eligibility (n=57)
Excluded (n=36)
• Not meeting inclusion criteria (n=24)
• Declined to participate (n=0)
• Other reasons (n=12)
Randomized (n=21)
Allocated to leptin (n=11)
• Received allocated intervention (n=11)
• Did not receive allocated intervention (n=0)
Allocated to placebo (n=10)
• Received allocated intervention (n=10)
• Did not receive allocated intervention (n=0)
Lost to follow-up (n=0)
Discontinued intervention (injection-site reactions) (n=1)
Lost to follow-up (n=1)
Discontinued intervention (n=0)
Analyzed (n=10)
• Excluded from analysis (n=0)
Analyzed (n=9)
• Excluded from analysis (n=0)

Supplementary Figure 7: Flow-diagrams of the four clinical trials

Supplementary Note: Consort Checklist

Section/Topic	Item No	Checklist item	Reported on page No
**Title and abstract**			
	1a	Identification as a randomised trial in the title	-
	1b	Structured summary of trial design, methods, results, and conclusions (for specific guidance see CONSORT for abstracts)	2
**Introduction**			
Background and objectives	2a	Scientific background and explanation of rationale	3-4
	2b	Specific objectives or hypotheses	3-4
**Methods**			
Trial design	3a	Description of trial design (such as parallel, factorial) including allocation ratio	17-21
	3b	Important changes to methods after trial commencement (such as eligibility criteria), with reasons	-
Participants	4a	Eligibility criteria for participants	17-21
	4b	Settings and locations where the data were collected	17-21
Interventions	5	The interventions for each group with sufficient details to allow replication, including how and when they were actually administered	17-21
Outcomes	6a	Completely defined pre-specified primary and secondary outcome measures, including how and when they were assessed	17-21
	6b	Any changes to trial outcomes after the trial commenced, with reasons	-
Sample size	7a	How sample size was determined	-
	7b	When applicable, explanation of any interim analyses and stopping guidelines	-
Randomisation:			
Sequence generation	8a	Method used to generate the random allocation sequence	-
	8b	Type of randomisation; details of any restriction (such as blocking and block size)	-
Allocation concealment mechanism	9	Mechanism used to implement the random allocation sequence (such as sequentially numbered containers), describing any steps taken to conceal the sequence until interventions were assigned	-
Implementation	10	Who generated the random allocation sequence, who enrolled participants, and who assigned participants to interventions	-
Blinding	11a	If done, who was blinded after assignment to interventions (for example, participants, care providers, those assessing outcomes) and how	-
	11b	If relevant, description of the similarity of interventions	-
Statistical methods	12a	Statistical methods used to compare groups for primary and secondary outcomes	25-28
	12b	Methods for additional analyses, such as subgroup analyses and adjusted analyses	25-28

Section/Topic	Item No	Checklist item	Reported on page No
**Results**			
Participant flow (a diagram is strongly recommended)	13a	For each group, the numbers of participants who were randomly assigned, received intended treatment, and were analysed for the primary outcome	Sup. Figure 7
	13b	For each group, losses and exclusions after randomisation, together with reasons	17-21 & Sup. Figure 7
Recruitment	14a	Dates defining the periods of recruitment and follow-up	-
	14b	Why the trial ended or was stopped	-
Baseline data	15	A table showing baseline demographic and clinical characteristics for each group	-
Numbers analysed	16	For each group, number of participants (denominator) included in each analysis and whether the analysis was by original assigned groups	17-21
Outcomes and estimation	17a	For each primary and secondary outcome, results for each group, and the estimated effect size and its precision (such as 95% confidence interval)	-
	17b	For binary outcomes, presentation of both absolute and relative effect sizes is recommended	-
Ancillary analyses	18	Results of any other analyses performed, including subgroup analyses and adjusted analyses, distinguishing pre-specified from exploratory	4-12
Harms	19	All important harms or unintended effects in each group (for specific guidance see CONSORT for harms)	-
**Discussion**			
Limitations	20	Trial limitations, addressing sources of potential bias, imprecision, and, if relevant, multiplicity of analyses	16
Generalisability	21	Generalisability (external validity, applicability) of the trial findings	15-16
Interpretation	22	Interpretation consistent with results, balancing benefits and harms, and considering other relevant evidence	12-15
**Other information**			
Registration	23	Registration number and name of trial registry	18, 19, 21
Protocol	24	Where the full trial protocol can be accessed, if available	-
Funding	25	Sources of funding and other support (such as supply of drugs), role of funders	33
