# Supplementary material for: Leptin alters energy intake and fat mass but not energy expenditure in lean subjects
Source: Nat Commun. 2020 Oct 13;11:5145. doi: 10.1038/s41467-020-18885-9 (PMC7553922; doi:10.1038/s41467-020-18885-9)
Supplement: Supplementary file 3 — Description of Additional Supplementary Files [file 41467_2020_18885_MOESM3_ESM.pdf]

Description of Additional Supplementary Files

Title: Supplementary Data 1

Description: Serum concentrations of lipoproteins, lipids and metabolites in study 1 (72hfed untreated or fasting treated with leptin or placebo) Data are presented as Mean ± SE. Mixed model was performed adjusted for baseline. P-values are presented for parameter “Group” i.e. Fed, Fasting+Placebo, Fasting+Leptin in mixed model; parameter “Time” i.e. 0,1,2,3 completed days of fasting; interaction of the two parameters. By p in mixed model (parameter group or group*time) <0.05, post-hoc Bonferroni test was performed between the estimated means of the three groups. “a” indicates p-value<0.05 for Fed vs Fasting+Placebo; “b” indicates p-value<0.05 for Fed vs Fasting+Leptin; “c” indicates p-value<0.05 for Fasting+Placebo vs Fasting+Leptin. "Processed" data set, as defined in statistical analysis section was used.

Title: Supplementary Data 2

Description: Description of all the identified metabolites included in this study
