# Supplementary material for: Leptin alters energy intake and fat mass but not energy expenditure in lean subjects
Source: Nat Commun. 2020 Oct 13;11:5145. doi: 10.1038/s41467-020-18885-9 (PMC7553922; doi:10.1038/s41467-020-18885-9)
Supplement: Supplementary file 6 — Reporting Summary [file 41467_2020_18885_MOESM6_ESM.pdf]

nature research

Corresponding author(s): Christos Mantzoros

Last updated by author(s): Sep 7, 2020

Reporting Summary

Nature Research wishes to improve the reproducibility of the work that we publish. This form provides structure for consistency and transparency in reporting. For further information on Nature Research policies, see our Editorial Policies and the Editorial Policy Checklist.

Statistics

For all statistical analyses, confirm that the following items are present in the figure legend, table legend, main text, or Methods section.

n/a	Confirmed	
☐	☒	The exact sample size (*n*) for each experimental group/condition, given as a discrete number and unit of measurement
☐	☒	A statement on whether measurements were taken from distinct samples or whether the same sample was measured repeatedly
☐	☒	The statistical test(s) used AND whether they are one- or two-sided<br>*Only common tests should be described solely by name; describe more complex techniques in the Methods section.*
☐	☒	A description of all covariates tested
☐	☒	A description of any assumptions or corrections, such as tests of normality and adjustment for multiple comparisons
☐	☒	A full description of the statistical parameters including central tendency (e.g. means) or other basic estimates (e.g. regression coefficient) AND variation (e.g. standard deviation) or associated estimates of uncertainty (e.g. confidence intervals)
☐	☒	For null hypothesis testing, the test statistic (e.g. *F*, *t*, *r*) with confidence intervals, effect sizes, degrees of freedom and *P* value noted<br>*Give P values as exact values whenever suitable.*
☒	☐	For Bayesian analysis, information on the choice of priors and Markov chain Monte Carlo settings
☒	☐	For hierarchical and complex designs, identification of the appropriate level for tests and full reporting of outcomes
☐	☒	Estimates of effect sizes (e.g. Cohen's *d*, Pearson's *r*), indicating how they were calculated

Our web collection on statistics for biologists contains articles on many of the points above.

Software and code

Policy information about availability of computer code

Data collection	No software was used
Data analysis	Data analysis was performed with SPSS v19.0 (SPSS, Inc,, Chicago, IL) for Windows, with GraphPad prism 7 (GraphPad Software Inc,, La Jolla, CA), with MetaboanalystR (Chong et el, 2018) and with RStudio ("rmcorr" package version 0.3.0).

For manuscripts utilizing custom algorithms or software that are central to the research but not yet described in published literature, software must be made available to editors and reviewers. We strongly encourage code deposition in a community repository (e.g. GitHub). See the Nature Research guidelines for submitting code & software for further information.

Data

Policy information about availability of data

All manuscripts must include a data availability statement. This statement should provide the following information, where applicable:

- Accession codes, unique identifiers, or web links for publicly available datasets
- A list of figures that have associated raw data
- A description of any restrictions on data availability

Source data are provided with this paper. Source data file includes data for Figures 2-7, Supplementary Figures 1-6, Supplementary Table 1 and Supplementary Data 1. Any other data that support the findings of this study are available from the corresponding author upon reasonable request.

April 2020

Field-specific reporting

Please select the one below that is the best fit for your research. If you are not sure, read the appropriate sections before making your selection.

☒ Life sciences ☐ Behavioural & social sciences ☐ Ecological, evolutionary & environmental sciences

For a reference copy of the document with all sections, see nature.com/documents/nr-reporting-summary-flat.pdf

Life sciences study design

All studies must disclose on these points even when the disclosure is negative.

Sample size	Study 1: Sample size was determined to 8-12 males and 8-12 females as described in the uploaded protocol. Sample size was based on previously published work and on power analysis alpha=5% and 1-β=80% to detect a 50% difference in mean leptin concentration.<br>Study 2: Sample size was determined to 5 subjects per group (5 lean males, 5 obese males, 5 lean females and 5 obese males) as described in the uploaded protocol. No previous pharmakokinetics studies involving subjects in the fasting state had been performed. Thus, no preliminary data were available and the selection of 5 subjects per group was made arbitrarily guided by our preliminary findings according to which lean men studied in the fed state had a mean leptin level of 2.1+/-0.4ng/ml which decreased to 0.4+/-0.3ng/ml during the fasted state. If the lean and obese subjects were pooled together (10 in each group), sample size based on power calculations alpha=5% and 1-β=80% would detect a difference of mean peak leptin concentration (Cmax) equal or larger to 1.5 times the respective SD. Studying five subjects per group would provide power 1-β=79% to detect a difference of mean Cmaz equal or larger than 2.0 the within group SD.<br>Study 3: Plan was to recruit 20 subjects with an aim to have 6 evaluable subjects at the end of the study as described in the uploaded protocol. Sample size was based on power analysis alpha=5% and 1-β=80% to detect a difference between an expected rate of spontaneous menstrual periods over 3 months of 5% vs a rate of 45% in the treatment group.<br>Study 4: The total number of evaluable subjects needed for the study was determined to 34 (17 per treatment arm). Plan was to screen and consent up to 100 subjects in order to have 50 subjects entering the study and 34 subjects completing the study as described in the uploaded protocol. Sample size was based on previously published work and on power analysis alpha=5% and 1-β=80% such that a minimum of 4% difference in bone mineral density could be detected between groups and assuming a variance model with SD=4. difference.
Data exclusions	Study 1: Six males and six females completed all three study phases/visits. Two males withdrew before completing all three phases, and one female before completing one of the phases (fasting + placebo). We excluded the two males from the analysis as their corresponding data was insufficient, but we included the female since she had completed 2/3 phases/visits, so that in total findings from 13 subjects (6 males and 7 females) were analyzed.<br>Study 2: Five normal weight men, five men with obesity and five normal weight women participated in all 6 admissions and were analyzed in our study. Recruitment in obese females was not completed and thus were not included in the study as stated in protocol.<br>Study 3: Eight females were initially enrolled, one subject withdrew after one month for reasons unrelated to the study, and thus, the results are derived from the remaining seven subjects.<br>Study 4: Among the 20 participants who were enrolled in the study, 11 were assigned randomly to receive metreleptin and 9 to receive placebo (age=25.4+/-1.2 years; BMI=19.8+/-0.7kg/m2). One participant in the metreleptin treated group withdrew from the study because she developed injection-site reactions soon after the baseline visit, leaving 10 in the metreleptin group and 9 in the placebo group.
Replication	All samples were run in duplicates within the same run for a given subject and were repeated if coefficient of variation for any sample was N15%. All attempts of replication were successful.
Randomization	Study 1: Subjects were assigned in a random order to each of the three admissions (i.e. fed, fasting+placebo, fasting+leptin)<br>Study 2: No specific method of randomization was used. The series of admissions started with the lowest dose moving to the highest dose for safety purposes. For fasting studies, subjects received dose A (0.01mg/kg) during Admission 1, dose B (0.1mg/kg) during Admission 2 and dose C (0.3mg/kg) during Admission 3. For fed studies, a similar protocol was followed. For males, fed studies were performed after the completion of all three fasting studies. For females, the first day of each fasting study was scheduled during the beginning of each follicular phase and thus fed studies were conducted either in between or after the fasting studies. The fed studies were scheduled as 3 separate 1 day admissions or as 2 admissions: a 2 day admission (Dose A on Day 1 and Dose B on Day 2) and a 1 day admission (Dose C).<br>Study 3: No method of randomization was necessary since this is a single-arm study.<br>Study 4: Subjects were randomly assigned in a 1:1 fashion to receive metreleptin or placebo.
Blinding	Study 1: Investigators and participants were blinded during group allocation and data collection (double blinded)<br>Study 2: Investigators or participants were not blinded. The series of admissions started with the lowest dose moving to the highest dose for safety purposes. Due to the designed specific admission series blinding was not possible.<br>Study 3: Blinding was not necessary since this is a single-arm study.<br>Study 4: Investigators and participants were blinded during group allocation and data collection (double blinded)

Reporting for specific materials, systems and methods

We require information from authors about some types of materials, experimental systems and methods used in many studies. Here, indicate whether each material, system or method listed is relevant to your study. If you are not sure if a list item applies to your research, read the appropriate section before selecting a response.

Materials & experimental systems

n/a	Involved in the study
☒	☐ Antibodies
☒	☐ Eukaryotic cell lines
☒	☐ Palaeontology and archaeology
☒	☐ Animals and other organisms
☐	☒ Human research participants
☐	☒ Clinical data
☒	☐ Dual use research of concern

Methods

n/a	Involved in the study
☒	☐ ChIP-seq
☒	☐ Flow cytometry
☒	☐ MRI-based neuroimaging

Human research participants

Policy information about studies involving human research participants

Population characteristics

Study 1: Eight healthy lean men (age=23.3±1.2yr; BMI = 23.7±0.6 kg/m2) and seven healthy lean women (age=22.4±1.2 yr; BMI= 21.7 ±2.2 kg/m2) with regular menstrual cycles and not on oral contraceptives for at least 6 months participated in the study.
Study 2: Five normal weight men (age=22.2±0.9yr; BMI=22.0±0.5 kg/m2), five men with obesity (age=23.4±1.5yr; BMI=32.0 ±1.0 kg/m2), and five normal weight women (age=20.4±0.7yr; BMI=21.9±0.7 kg/m2) participated in the 6 admissions.
Study 3: Eight lean women (age=24.8±5.4 years; body mass index (BMI) =20.5±2.0 kg/m2) with acquired hypoleptinemia due to secondary hypothalamic amenorrhea (HA from strenuous exercise, without active eating disorders and with stable weight (inclusion criteria: within±15% of ideal bodyweight for ≥ six months) participated in the study.
Study 4: Twenty females, between 18 and 35 years old with hypoleptinemia due to secondary HA for ≥ 6 months coincident with strenuous exercise and/or low body weight (within±15% of ideal bodyweight for ≥ six months at the time of screening) participated in the study and were randomized with a 1:1 allocation to receive either metreleptin or placebo.

Recruitment

Study 1: Healthy individuals were recruited from the Boston area using advertisement and postings through the Boston area Universities Students Offices. Subjects were recruited in proportion to their ethnic balance in the local community. Subjects were chosen without regard for racial, social, economic or other status.
Study 2: Healthy individuals were recruited from the Boston area using advertisement and postings through Boston area universities' students offices. Subjects were recruited in proportion to their ethnic balance in the local community. Subjects were chosen without regard for racial, social, economic or other status.
Study 3: Up to twenty healthy, normal-weight women with hypothalamic amenorrhea and amenorrheic women athletes were recruited from the community as well as the Endocrine clinics at Beth Israel Deaconess Medical Center or Massachusetts General Hospital, without regard for racial, social, economic or other status.
Study 4: Healthy, normal-weight women with hypothalamic amenorrhea were recruited through BIDMC and possibly other centers such as Children's Hospital, and Columbia University. Subjects were recruited without regard for racial, social, economic, or other status.

Ethics oversight

Institutional Review Board of the General Clinical Research Center (GCRC) of the Beth Israel Deaconess Medical Center

Note that full information on the approval of the study protocol must also be provided in the manuscript.

Clinical data

Policy information about clinical studies

All manuscripts should comply with the ICMJEguidelines for publication of clinical research and a completedCONSORT checklist must be included with all submissions.

Clinical trial registration

ClinicalTrials.gov Identifier
Study 1: NCT00140231
Study 2: NCT00140205
Study 4:NCT00130117

Study protocol

Study protocols are uploaded in figshare.com.
This is the private link to the protocols
https://figshare.com/s/696fe9847bc6898de577

Data collection

Study 1: Subjects were be admitted to the GCRC for a 4 day admission
Blood draw was performed in the evening of day 0 and in the morning of days 1,2,3 prior to any intervention. RMR was measured in the morning (7:00-8:00am) of days 1 and 3/4. Body composition measurements were performed in the morning of days 1 and 4 (between 7:30-9:30am). 24-hr urine sampling was performed on day 3 starting at 8:00-8:30am/

Study 2: Each participant participated in 3 fed-normoleptinemic and 3 fasting-induced hypoleptinemic studies, which were conducted in the GCRC. The duration of each fasting-induced hypoleptinemic visit was 72 hours. The duration of each fed/normoleptinemic study was 24 hours. Vital signs were measured at 7:00, 14:00 and 18:00-20:00 of each study day. Leptin was administered at 8:00 every morning. Leptin levels were measured at +30 minutes, +1hr, + 2hr, +3hr, +4hr, +5hr, +6hr, +8hr, +10hr, +12hr, +18hr, and +24hr after each dose. Serum samples obtained in the early morning (prior to dose administration), noon (after leptin's peak) and evening of each study day were used for renin, aldosterone, FFA and lipoprotein/metabolite (NMR) measurements. For fatty acids

with GC-MS, serum samples were examined on Day 1 at 8:00 (before leptin administration), and on Days 1, 2 and 3 at 14:00(close to the serum peak of leptin) were available. Finally, urine catecholamines were measured at baseline and on day 3 of each admission at the fasting state. Renin, aldosterone and urine catecholamine measurements were not available in the fed state.

Study 3: Blood samples were obtained weekly and body composition was determined with dual-energy X-ray absorptiometry (DEXA) every other week, starting one month before initiation of leptin treatment (baseline month, where measurements were performed at the beginning and end of the month). RMR was measured (DeltraTrac II Metabolic Monitor, SensorMedics) during the baseline month and after 15 days of leptin treatment. Daily exercise records were obtained. For all visits, subjects were admitted to the GCRC.

Study 4: Fasting blood samples were collected every four weeks, while body composition and RMR were measured every 12 weeks with DEXA and Sensormedics Vmax Encore equipment (VIASYS Respiratory Care Inc,), respectively. For all visits, subjects were admitted to the GCRC.

Outcomes

The primary and secondary outcomes of the studies have been previously described in the uploaded protocols.
The primary outcomes of our analysis were a) correlations of baseline leptin levels with % of weight change in all four clinical studies – interventions, b) differences in % weight changes between lean men, lean women and obese in escalating leptin doses, c) weight, fat mass and FFA changes in relation to leptin levels during long-term leptin treatment and after its termination. The secondary outcomes were changes in energy expenditure (i.e. RMR and physical activity), energy intake, SNS activity (i.e. HR, BP, body temperature, serum/urine catecholamines) , and metabolic profile (i.e. lipoproteins, amino acids, fatty acids, ketone bodies) in all four clinical studies-interventions.
