# Supplementary material for: Leptin alters energy intake and fat mass but not energy expenditure in lean subjects
Source: Nat Commun. 2020 Oct 13;11:5145. doi: 10.1038/s41467-020-18885-9 (PMC7553922; doi:10.1038/s41467-020-18885-9)
Supplement: Supplementary file 2 — Peer Review File [file 41467_2020_18885_MOESM2_ESM.pdf]

Editorial Note: This manuscript has been previously reviewed at another journal that is not operating a transparent peer review scheme. This document only contains reviewer comments and rebuttal letters for versions considered at *Nature Communications*.

Reviewers' Comments:

Reviewer #3:
Remarks to the Author:
Reviewer critiques have been adequately addressed.
